# Unlocking the potential of Bi_2_S_3_ for photocatalysis: a roadmap for next-generation solar catalysts

**DOI:** 10.1039/d6sc01361a

**Published:** 2026-04-17

**Authors:** Wei Zhao, Qing Chen, Lifeng Cai, Jie Liang, Fang-Xing Xiao

**Affiliations:** a Key Laboratory of Ecological Environment and Information Atlas, Fujian Provincial University (Putian University), College of Environmental and Biological Engineering Putian 351100 P. R. China 89437499@qq.com; b Fujian Key Laboratory of Ecological Impacts and Treatment Technologies for Emerging Contaminants, Putian University Putian 351100 P. R. China ptxylj0321@126.com; c College of Materials Science and Engineering, Fuzhou University New Campus Minhou Fujian Province 350108 P. R. China fxxiao@fzu.edu.cn

## Abstract

Photocatalysis enables the direct conversion of solar energy into chemical fuels, presenting a compelling strategy to mitigate the global energy crisis and environmental pollution. However, traditional photocatalysts are severely hampered by inefficient visible-light harvesting and undesirably rapid recombination of photogenerated carriers, which bottlenecks their large-scale practical deployment. Thus, developing efficient, stable, and broadband-responsive photocatalytic materials remains a paramount research imperative. Bismuth sulfide (Bi_2_S_3_), a prototypical narrow-bandgap semiconductor, has recently garnered immense interest. Its judiciously positioned band edges and strong visible-light absorption confer distinct advantages for solar-driven photoredox reactions. Despite significant advances, the field still lacks a comprehensive and timely review consolidating Bi_2_S_3_-based artificial photosystems. This review systematically summarizes the latest progress in Bi_2_S_3_-based photocatalysts, with a particular focus on morphology control, heterojunction construction, elemental doping, and defect engineering. We elucidate how these strategies precisely manipulate the electronic structure, facilitate charge separation, broaden light absorption, and enhance material stability. Furthermore, we outline critical future perspectives: (i) designing novel multicomponent architectures, (ii) unraveling the kinetic mechanisms of interfacial carrier transfer, and (iii) validating scalable performance under realistic environmental conditions. This review provides a holistic roadmap for Bi_2_S_3_-mediated photoredox catalysis, serving as a vital resource for researchers advancing solar energy conversion technologies.

## Abstract 2

The latest developments of Bi_2_S_3_-based artificial photosystems in multifarious photoredox catalysis are elucidated with future perspectives elucidated.

## Graphical abstract

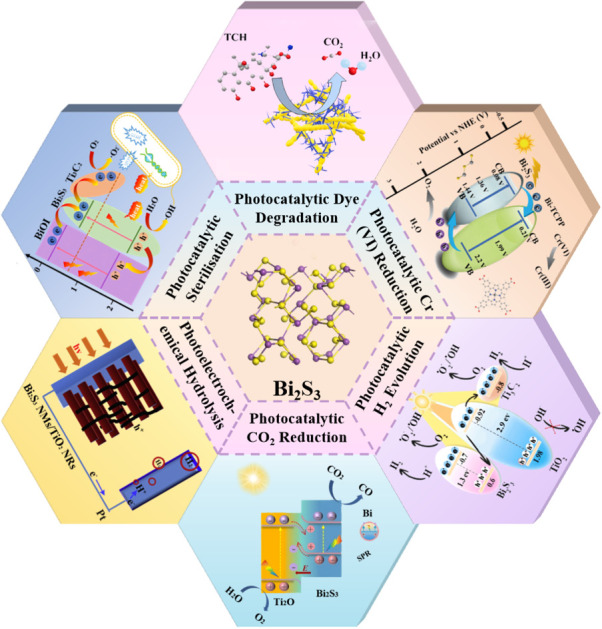

## 1. Introduction

Photocatalysis offers a unique advantage in directly converting solar energy into chemical energy, showing broad prospects for addressing energy shortages and environmental remediation.^1–3^ The core of photocatalysis involves photo-exciting semiconductors to utilize the electrons and holes for redox reactions. This fundamental process relies heavily on semiconductor materials absorbing photon energy to create electro–hole pairs, which then drive a series of redox reactions such as photocatalytic H_2_ production, CO_2_ reduction, mineralization of organic pollutants, and bacterial disinfection.^4–7^ This process mainly consists of three consecutive yet interrelated key stages, which includes photon absorption and carrier excitation, charge separation and migration, and surface redox reactions.

Despite its great potential, photocatalysis still faces several technical hurdles before large-scale deployment. First, the separation efficiency of photogenerated charge carriers is intrinsically low. Most electron–hole pairs recombine within nanoseconds to picoseconds after formation, severely limiting the quantum yield. Thus, low quantum efficiency and severe carrier recombination remain long-standing bottlenecks for photocatalytic materials.^8^ Second, conventional photocatalysts such as TiO_2_ absorb only UV light, which accounts for less than 5% of the solar spectrum, leading to poor overall solar energy utilization.^9^ Moreover, current photocatalytic materials still exhibit shortcomings in long-term reaction stability, raw-material cost, and scalable preparation technologies,^10^ which further restrict their practical application scope.

The central challenge in photocatalysis is to harvest solar energy efficiently while boosting redox reaction efficiency. To this end, researchers have devoted sustained effort to developing new photocatalysts, such as metal oxides, metal sulfides, and plasmonic metal nanocrystals. Representative metal oxides such as TiO_2_ and ZnO exhibit good stability and low cost, yet their wide band gaps restrict absorption mainly to the UV region, leaving visible light largely unutilized.^11–13^ Although graphitic carbon nitride (g-C_3_N_4_), as a non-metallic semiconductor, offers visible-light activity and high chemical stability, its limited surface area and rapid carrier recombination hinder further enhancement of catalytic performance.^14^ Narrow-band-gap sulfides such as CdS exhibit strong visible-light absorption, yet suffer from severe photocorrosion and potential environmental toxicity.^15^ Overall, conventional photocatalysts still fail to overcome the simultaneous bottlenecks of high carrier-recombination rate, restricted solar-spectral response, and insufficient long-term stability. Against this backdrop, bismuth sulfide (Bi_2_S_3_), a V–VI group narrow-band-gap semiconductor, has emerged as a research hotspot owing to its unique structure and optoelectronic properties along with low toxicity, offering a fresh strategy to break the above bottlenecks and driving diverse photocatalytic reactions including CO_2_ reduction, N_2_ fixation and heavy-metal reduction. The band gap of Bi_2_S_3_ can be tuned between 1.3 and 1.7 eV, pushing its absorption edge into the near-infrared (*ca.* 800–1000 nm) and covering roughly 40% of the solar energy spectrum. Its high absorption coefficient (10^4^–10^5^ cm^−1^) endows it with exceptional light-harvesting capability. Moreover, a favorable combination of high carrier mobility and suitable band-edge positions facilitates the efficient charge separation and accelerates charge migration to surface reaction sites, conferring significant advantages in light-conversion kinetics.^16^

However, inherent drawbacks such as short carrier-diffusion lengths and facile photocorrosion under illumination still restrict the practical deployment of Bi_2_S_3_.^17^ To address these issues, a variety of modification strategies have been developed including morphology control (quantum dots, nanorods, nanosheets),^18^ heterojunctions engineering (Type-II, Z-scheme or S-scheme),^19–21^ elemental doping (Fe, N, *etc.*),^22^ single-atom deposition,^23^ sulfur vacancies modulation,^23,24^ and hybridizing with cocatalysts (MoS_2_, NiS).^25^ These modified strategies provide alternative approaches to accelerate the charge transport kinetics, increase specific surface area, and boost charge separation efficiency of Bi_2_S_3_-based artificial photosystems.^26^ Despite the advancement, there is still lack of timely, comprehensive, and systematic review article that summarizes the latest development of Bi_2_S_3_-based artificial photosystems.

In this review, we present a comprehensive 2019–2025 roadmap of Bi_2_S_3_-based photocatalysis following a “structure–property-application” hierarchy. Starting with its intrinsic crystal and electronic traits, we summarize the precision-synthesis tools (hot-injection, template, microwave, *etc.*) and dissect the morphology-performance links across dimensions from 0D quantum dots to 3D flower-spheres. We then spotlight Type-II, Z-scheme, p–n and Schottky heterojunctions, coupled with doping, defect and cocatalyst synergies, and showcase their emerging application in photocatalytic pollutant degradation, CO_2_ reduction, N_2_ fixation, photoelectrochemical (PEC) H_2_ evolution and bacterial disinfection. The future perspectives and challenges are finally provided for further pushing forward the prosperity of Bi_2_S_3_-based photocatalysis toward solar energy conversion.

## 2. Fundamental properties of Bi_2_S_3_

### 2.1. Crystal and electronic structure of Bi_2_S_3_

#### 2.1.1. Lattice structure

**Fig. 1:**
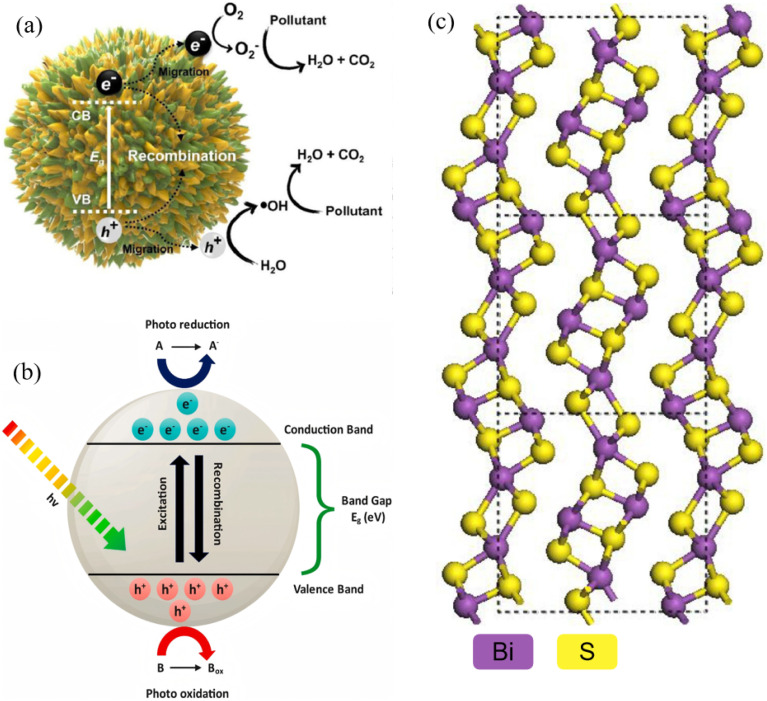
(a) Photocatalytic mechanism diagram.^29^ Copyright 2023, Elsevier Ltd. (b) Schematic representation of mechanisms involved in semiconductor-based photocatalysis.^2^ Copyright 2024, Elsevier Ltd. (c) Bi_2_S_3_ crystal structure and band structure.^16^ Copyright 2025, Elsevier.

Bi_2_S_3_ crystallizes in an orthorhombic system. Kyono *et al.*, first identified the natural mineral as the orthorhombic stibnite structure (space group *Pnma*) by single-crystal diffraction, with lattice constants *a* ≈ 11.15 Å, *b* ≈ 11.30 Å, *c* ≈ 3.98 Å and 20 atoms per unit cell (4 formula units of Bi_2_S_3_).^27^ The architecture is strongly anisotropic, that is, the fundamental motif is an infinite (Bi_2_S_3_)_*n*_ ribbon that propagates in a zig-zag fashion along the *c*-axis. Within each ribbon Bi is octahedrally coordinated and S is approximately close-packed with every Bi atom bonding to seven S atoms, thereby giving two distinct short (2.589–2.738 Å, covalent) and long (2.975–3.328 Å, van-der-Waals-like) bond lengths (Fig. 1). Adjacent ribbons are linked only by these weak Bi–S interactions, yielding a highly anisotropic layered array. Because the lowest surface energy is along the chain direction, crystals grow preferentially into one-dimensional nanowires or nanorods, providing a structural basis for morphology control.^28^

This orthorhombic lattice is thus a hybrid chain-layer structure whose hallmark is “infinite Bi_2_S_3_ chains aligned along [001]”. Black *et al.*, using early X-ray crystallography, showed that Bi^3+^ and S^2−^ form endless chains parallel to the *c*-axis. Specifically, Bi^3+^ occupies a strongly distorted octahedron in which three short covalent Bi–S bonds stabilize the chain and dictate the preferential growth along *c*.^30^ Consequently, bonding is covalent and strong along *c*, whereas the inter-chain interactions perpendicular to the *a*–*b* plane are weak van-der-Waals forces. Researchers investigating nanostructure growth emphasize that this bond hierarchy is the key to the facile formation of 1-D nanomorphologies. In other words, rapid *c*-axis growth is driven by strong covalent bonds, while lateral growth is kinetically hindered by weak inter-chain forces.^31^ The lattice anisotropy directly governs electronic and optical behaviour. Deshpande *et al.*, observed a blue-shifted absorption edge in Bi_2_S_3_ nanorods and attributed it to the combined effects of quantum confinement and the oriented chain-like atomic arrangement, which modifies the electronic transition barrier.^32^

#### 2.1.2. Electronic band structure

**Fig. 2:**
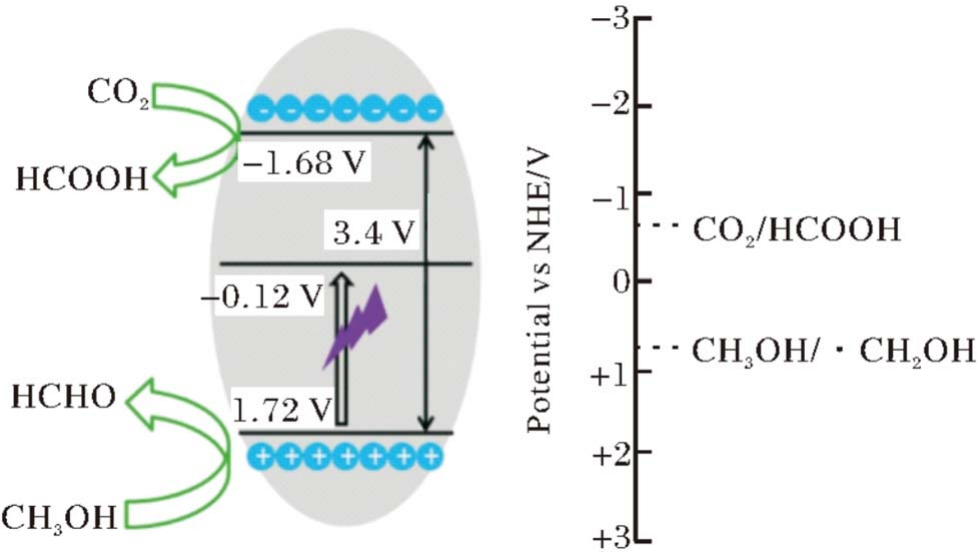
Band structure of Bi_2_S_3_ microspheres.^37^ Copyright 2013, Royal Society of Chemistry.

The narrow band gap of Bi_2_S_3_ (1.3–1.7 eV) (Fig. 2) is the fundamental reason that it can harvest visible-to-near-infrared photons with high efficiency.^16^ Density functional theory (DFT) provides an atomistic roadmap for understanding both the gap origin and the catalytic activity of Bi_2_S_3_. First-principles calculations show that the valence-band maximum (VBM) originates from hybridized S 3p and Bi 6s orbitals. Because Bi is a heavy element, its 6s level is pushed upward by strong spin orbit coupling (SOC), raising the VBM and thus shrinking the gap. The conduction-band minimum (CBM) is dominated by Bi 6p states, endowing the material with a powerful reduction potential.^33–36^ This unique p–s orbital mixing, amplified by SOC, produces the characteristic narrow gap. The DFT calculations start from the intrinsically SOC-narrowed band gap, proceed through strain fine-tuning, and culminate in interfacial charge reorganization, providing a reliable theoretical tool for interpreting band-gap engineering and heterostructure construction.

### 2.2. Optical and electrical properties of Bi_2_S_3_

#### 2.2.1. Light-absorption characteristics

The intrinsic narrow band gap and high density of p–s antibonding states at the valence-band top endow Bi_2_S_3_ with broadband visible-to-near-infrared (NIR) harvesting. Its absorption edge extends to 800–1000 nm, and the absorption coefficient in the visible region reaches 10^4^–10^5^ cm^−1^. Recent work has disentangled how size, heterojunctions and local electromagnetic fields jointly tailor the absorption edge, coefficient and hot-electron yield. For example, Li *et al.*^38^ deposited 6–8 nm Bi_2_S_3_ quantum dots (QDs) on TiO_2_ nanotubes by a SILAR (Successive Ionic Layer Adsorption and Reaction) method. UV-vis diffuse reflectance showed that bulk-like Bi_2_S_3_ absorbs up to 800 nm (1.3 eV), whereas the Bi_2_S_3_/TiO_2_ QD film exhibits a blue-shifted edge at 780 nm (1.59 eV). The average absorption coefficient in the 400–800 nm window reaches 1.2 × 10^5^ cm^−1^ which is five times that of bare TiO_2_ (2.4 × 10^4^ cm^−1^), demonstrating that QD confinement preserves high absorbance while enabling gap tunability. When the QD film was used as a photoanode, photogenerated electrons were injected into stainless steel, lowering its potential and preventing corrosion. This solar-driven cathodic protection strategy directly evidences the synergistic high-absorption and efficient-carrier-output feature of Bi_2_S_3_ QDs. Chen *et al.*^39^ successfully prepared a Bi_2_S_3_/ReS_2_ heterojunction. Coupling with ReS_2_ synergistically exploits the narrow band gaps of both components, broadening the light-harvesting range and enhancing absorption intensity. Moreover, tuning the Bi_2_S_3_/ReS_2_ mass ratio significantly boosts NIR absorption efficiency. This superior optical absorption underpins efficient photothermal conversion (35.2% efficiency in aqueous solution) and Z-scheme charge transfer, ultimately elevating photocatalytic H_2_ evolution performance to 7.36 times that of bare Bi_2_S_3_. In another case, Jiang *et al.*^40^ synthesized Bi_2_S_3_ nanorods with defect structures *via* a solvothermal route. UV-vis-NIR spectroscopy revealed that these nanorods exhibit a broad and intense absorption band in the near-infrared region (700–1400 nm) with a molar extinction coefficient of 12.3 L g^−1^ cm^−1^, significantly higher than conventional Bi_2_S_3_ nanomaterials. This absorption characteristic originates from the unique morphology and defect structure of the nanorods, wherein the defect structure confers metallic-like absorption properties, while the special morphology modulates the absorption peak position. Their synergy enhances NIR harvesting efficiency, enabling excellent photothermal conversion under 808 nm laser irradiation. This study confirms that the optical absorption properties of Bi_2_S_3_ nanorods can be tuned through morphology and defect engineering. Collectively, size, defect and morphology engineering can synchronize ultra-broad absorption (400–1200 nm) with efficient photothermal/carrier generation, laying the optical foundation for full-spectrum photocatalysis.

#### 2.2.2. Carrier dynamics

**Fig. 3:**
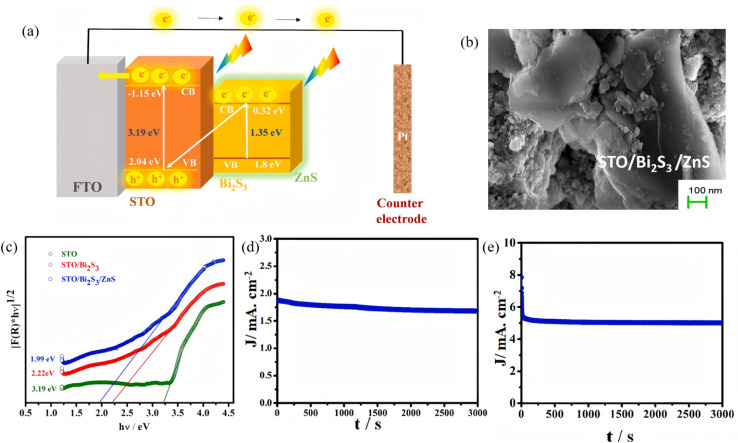
(a) Schematic representation of the band alignment and mechanism of electron transfer in the STO/Bi_2_S_3_/ZnS system. (b) SEM image of the STO/Bi_2_S_3_/ZnS composites. (c) Kubelka–Munk plot of reflectance spectra for band gap estimation. Chronoamperometric (*I*–*t*) curve of STO/Bi_2_S_3_/ZnS photoelectrode in (d) neutral medium (pH 7), (e) alkaline medium (pH 12.4).^41^ Copyright 2024, Elsevier.

Although the narrow band gap and high absorption coefficient of Bi_2_S_3_ guarantee efficient harvesting of visible-to-NIR photons, its intrinsic carrier diffusion length is short (∼100 nm) and recombination is rapid (*τ* < 10 ns), giving rise to pronounced bulk and surface losses that severely limit the quantum efficiency of photocatalysis. Recent studies have adopted a hierarchical strategy to unravel and manipulate charge separation and transport in heterostructures. Joy *et al.*^41^ deposited an atomic-layer-deposited ZnS passivation layer on SrTiO_3_/Bi_2_S_3_ nanorods to construct a ternary SrTiO_3_/Bi_2_S_3_/ZnS photoanode (Fig. 3). PL spectroscopy revealed that the ZnS layer isolates Bi_2_S_3_ from direct contact with the electrolyte while providing active states that promote charge transfer, markedly suppressing carrier recombination. The structure delivers a photocurrent density of 1.89 mA cm^−2^ in neutral medium and 5.06 mA cm^−2^ in alkaline medium, with a HC-STH (Half-Cell Solar-to-Hydrogen Efficiency) efficiency up to 4.8%, confirming the beneficial role of ZnS passivation in optimizing carrier kinetics. Xiao *et al.*^42^ designed a self-supporting B-g-C_3_N_*x*_/Bi_2_S_3_/CdS dual S-scheme heterojunction film. TRPL (Time-Resolved Photoluminescence) measurements showed significantly longer carrier lifetimes for this ternary architecture compared to binary heterojunctions or bare B-g-C_3_N_*x*_. The dual built-in electric fields of the double S-scheme provide multi-channel charge-transfer pathways that effectively suppress charge recombination, achieving a photocatalytic H_2_-evolution rate of 4.78 mmol g^−1^ h^−1^ and 98.7% oxytetracycline degradation. In summary, hierarchical synergy can prolong carrier lifetime, enable high-velocity shunting and achieve spatial separation in Bi_2_S_3_-based photosystems, laying the kinetic foundation for efficient full-spectrum photocatalysis.

## 3. Controllable synthesis of Bi_2_S_3_ nanomaterials

### 3.1. Synthesis method

#### 3.1.1. Hydrothermal/solvothermal methods

**Fig. 4:**
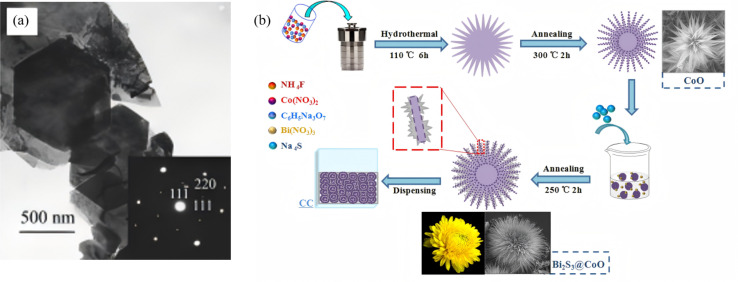
(a) TEM images of Bi_2_S_3_ obtained by solvothermal treatment at 160 °C for 48 h. Inset: ED patterns of the particles.^43^ Copyright 2003, Wiley-Blackwell. (b) Schematic illustration of the preparation process of 3D CoO and Bi_2_S_3_@CoO chrysanthemums-like arrays.^44^ Copyright 2019, Elsevier.

Hydrothermal/solvothermal synthesis has become the dominant route for the controlled fabrication of one-to three-dimensional Bi_2_S_3_ nanostructures, thanks to its simple equipment, low temperature, high crystallinity, and easily tunable morphology. Fundamentally, it couples reactions inside a sealed autoclave; by adjusting solvent polarity, temperature, and reaction time, the nucleation rate and facet-selective growth kinetics of Bi_2_S_3_ can be precisely tuned, enabling the targeted construction of ultra-long wires, nanobelts, or hierarchical nanoflowers. In 2003, Liu *et al.*^43^ first reported a classic solvothermal route for large-scale synthesis of Bi_2_S_3_ single-crystal nanobelts (Fig. 4a). Using Bi(NO_3_)_3_·5H_2_O and Na_2_S_2_O_3_ as precursors, with an aqueous NaOH/glycerol mixed solvent (volume ratio 2 : 1), reaction at 160 °C for 20 h *via* a solid-dissolution-solid transformation yielded single-crystal nanobelts 50–300 nm in width, 20–80 nm in thickness, and several millimeters in length, with high purity and yield. This work not only validated the feasibility of the “glycerol–NaOH–sulfur source” system, but also confirmed *via* HRTEM that nanobelts grow along [210] with side facets exposing (220) and (120) planes, clarifying the critical roles of glycerol coordination and NaOH concentration in dimension and facet control and providing essential theoretical foundations for subsequent Bi_2_S_3_ dimensional engineering and selective facet growth. As high specific surface areas and well-defined interfaces are beneficial for boosting the photoactivity, researchers have turned their attention to three-dimensional flower-ball structures self-assembled from two-dimensional nanosheets. For instance, Yang *et al.*^44^ employed solvothermal self-assembly followed by wet impregnation-annealing method to prepare 3D flower-like CoO using Co(NO_3_)_2_·6H_2_O as the cobalt precursor, and the wire-like micro-petals of CoO serve as an excellent growth substrate for Bi_2_S_3_. After wet impregnation and annealing, Bi_2_S_3_ nanosheets epitaxially grow on the CoO micro-petal surface, forming 3D chrysanthemum-like Bi_2_S_3_@CoO heterojunction arrays with an average size of ∼10.73 µm (Fig. 4b). The Bi_2_S_3_ nanosheets are ∼10 nm thick and form a stable heterointerface with the CoO (111) plane. This hierarchical structure combines high specific surface area, multiple light reflection/scattering channels, and optimized molecular diffusion kinetics, providing a structural foundation for efficient separation and migration of photogenerated charges in Z-scheme heterojunctions, thereby enhancing photocatalytic oxidation/reduction performance. Sang *et al.*^45^ proposed a pre-oxidation followed by *in situ* sulfidation one-step hydrothermal strategy. Bi(NO_3_)_3_·5H_2_O was dissolved in a DMF solution containing nitric acid, then mixed with an aqueous thiourea solution and transferred to an autoclave for reaction at 100 °C for 12 h. Under acidic conditions, thiourea slowly hydrolyzes to release S^2−^, which first reacts with Bi^3+^ to form a Bi_2_O_3_ nanosheet framework, followed by *in situ* sulfidation to yield Bi_2_O_3_/Bi_2_S_3_ p–n heterojunction flower-balls. The structure retains intimate contact between the Bi_2_O_3_ (111) and Bi_2_S_3_ (101) planes, establishing a built-in electric field that markedly suppresses photogenerated carrier recombination. Under visible light, 99.7% RhB is removed and 91.8% Cr(vi) is reduced within 90 min, and this outstanding performance stems from the synergistic effect of charge separation by the p–n heterojunction and the bifunctional catalytic sites. Recently, Wang *et al.*^46^ synthesized a Bi_2_S_3_–In_2_S_3_ heterostructure *via* a one-step hydrothermal route at 200 °C for 24 h, achieving face-to-face coupling between the Bi_2_S_3_ (060) and In_2_S_3_ (440) planes. In this architecture, Bi_2_S_3_ exists as nanorods (∼200 nm long, ∼20 nm in diameter) and In_2_S_3_ as nanoparticles (∼25 nm). The core of this facet coupling lies in the matching atomic spacing between the two phases, providing a robust interface for charge transfer. This face-to-face coupling not only shortens carrier diffusion distances but also delivers 100% Cr(vi) photoreduction within 30 min, which is about 44 times higher than bare Bi_2_S_3_, demonstrating a novel concept combining facet engineering with Z-scheme heterojunction optimization, and directly confirming the advantage of facet-to-facet coupling, shortening carrier diffusion distance while preserving highly active crystal planes.

From 1D single-crystalline nanobelts to 3D facet-coupled heteroflowers, hydrothermal/solvothermal methods exploit a three-parameter (solvent-ligand-temperature) coupling to achieve hierarchical control of dimension, facet, and band structure within a single reaction system. All products retain the intrinsic orthorhombic lattice of Bi_2_S_3_ while exposing different high-activity facets, offering a rich structural platform for subsequent photo and electro-catalytic optimization.

#### 3.1.2. Hot-injection method

**Fig. 5:**
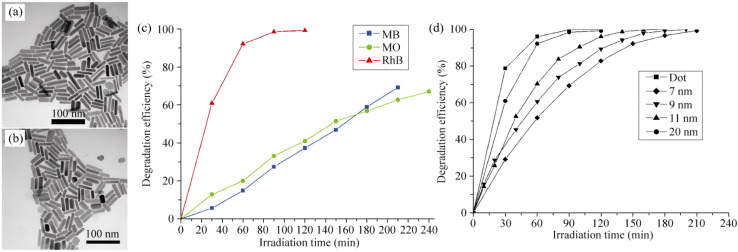
(a) TEM image of Bi_2_S_3_ nanocrystals prepared with a Bi/S ratio of 1 : 1.5 and Bi precursor concentrations of 0.025 mol L^−1^. (b) TEM images of Bi_2_S_3_ nanocrystals prepared with a Bi precursor concentration of 0.025 mol L^−1^ with Bi/S molar ratios of 1 : 1.5. (c) Photodegradation efficiency of Bi_2_S_3_ nanorods (prepared with a Bi source concentration of 0.025 mol L^−1^, Bi : S = 2 : 3) with MO, MB, and RhB. (d) Efficiency of Bi_2_S_3_ nanodots and nanorods with different diameters on the photodegradation of RhB.^47^ Copyright 2010, Tsinghua University Press.

The hot-injection (HI) technique is the cornerstone for producing monodisperse and highly crystalline Bi_2_S_3_ nanocrystals. By separating nucleation (high-temperature, seconds) from growth (lower-temperature, minutes), HI achieves temporal–spatial decoupling that yields quantum dots or nanorods with narrow size distribution and uniform morphology. As early as 2010, Wu *et al.*^47^ pioneered a hot-injection route to Bi_2_S_3_ nanostructures, wherein BiCl_3_ was dissolved in oleylamine at 150 °C, followed by rapid injection of a thioacetamide-oleylamine solution and heating to 180 °C for 5–10 min, yielding orthorhombic single-crystal nanorods (Fig. 5). The work confirmed that intrinsic chain-like growth along [001] drives the one-dimensional morphology. The resulting nanodots exhibited excellent visible-light photocatalytic activity due to their high specific surface area, efficiently degrading organic dyes such as RhB. To address the issues of poor mixing in dual-solution systems and poor batch-to-batch reproducibility, Saah *et al.*^48^ proposed a single-source-precursor-mediated hot-injection strategy, preparing high-purity orthorhombic Bi_2_S_3_ nanorods within 30 min. By co-injecting lead piperidine dithiocarbamate precursors, continuous synthesis of Pb_*x*_Bi_(1−*x*)_S alloy nanomaterials (Bi doping 0–100%) was achieved, with morphology evolving from cubic (low Bi, ≤50%) to rod-like (high Bi, ≥80%). The band gap tunes linearly from 0.72 eV (PbS) to 1.94 eV (Bi_2_S_3_). This single-source hot-injection method offers high atom economy (90–100% utilization), high product purity, and controllable morphology, providing high-performance model materials for optoelectronic devices and photovoltaics.

In summary, hot injection, based on millisecond supersaturation and second-scale nucleation, coupled with rational precursor design and the three-lever control strategy, enables continuous tuning of size, shape and hetero-interfaces of Bi_2_S_3_ nanocrystals. Its excellent reproducibility, high yield and straightforward scalability have made HI one of the preferred routes for both laboratory research and commercial production of high-quality Bi_2_S_3_ nanomaterials.

#### 3.1.3. Ion-exchange strategy

**Fig. 6:**
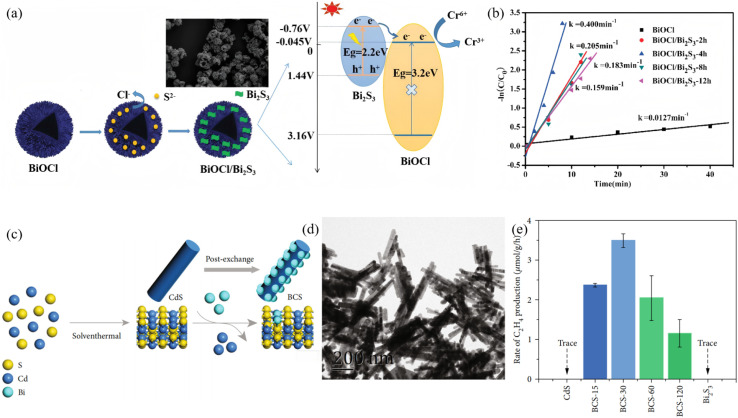
(a) Synthesis process and SEM images of hollow spherical BiOCl/Bi_2_S_3_, and photocatalytic mechanism of BiOCl/Bi_2_S_3_. (b) Plots of ln (C/C_0_) *versus* time for Cr^6+^ reduction over different catalysts.^49^ Copyright 2020, Elsevier. (c) Schematic illustration of the synthetic process of BCS–*t* composite. (d) TEM image of the BCS–30. (e) The yield of C_2_H_4_ from CdS, Bi_2_S_3_, and BCS–*t* under visible light irradiation.^50^ Copyright 2022, AMER ASSOC ADVANCEMENT SCIENCE.

Ion exchange, occurring at solid–liquid or solid–solid interfaces under ambient conditions, offers a mild, low-energy post-synthetic pathway for precisely tailoring Bi_2_S_3_-based heterojunctions. A highly reactive parent framework is first prepared, and subsequent selective replacement of anions (or cations) by S^2−^ and Bi^3+^ then produces an intimately bonded heterostructure in which band alignment and defect passivation are achieved simultaneously. Lu *et al.*^49^ employed hollow spherical BiOCl as a parent material and reacted it with thioacetamide (TAA) solution at room temperature. The hydrolysis of TAA releases S^2−^ ions that selectively replace Cl^−^ in BiOCl, generating Bi_2_S_3_ nanoparticles *in situ* on the BiOCl surface to form a BiOCl/Bi_2_S_3_ heterojunction (Fig. 6). XRD, XPS, SEM and TEM characterization confirmed that the composite fully retains the hollow spherical morphology of the BiOCl parent, with Bi_2_S_3_ nanoparticles uniformly dispersed on the surface. The Bi_2_S_3_ loading can be tuned by adjusting the ion-exchange reaction time. The resulting composite exhibits excellent Cr^6+^ reduction performance under visible light. The optimal sample BiOCl/Bi_2_S_3_-4h achieves a reduction rate constant of 0.400 min^−1^, completely reducing Cr^6+^ within 9 min, which is about 31 times higher than pure BiOCl. This anion-exchange strategy enables efficient heterointerface construction, providing a reference for the preparation of narrow-band-gap coupled photocatalytic systems. Huang *et al.*^50^ first synthesized CdS nanorods *via* solvothermal route, then used Bi^3+^-oleate complex as an exchange agent to exploit the much lower solubility product of Bi_2_S_3_ (*K*_sp_ ≈ 1 × 10^−97^) compared to CdS (*K*_sp_ ≈ 8 × 10^−27^), partially replacing Cd^2+^ with Bi^3+^ to disperse Bi_2_S_3_ nanoparticles *in situ* on CdS, forming a Bi_2_S_3_/CdS heterojunction. Mott–Schottky analysis confirmed that both CdS and Bi_2_S_3_ are n-type semiconductors, forming a Z-scheme band alignment. This core–shell cascade energy-level strategy drives photogenerated electrons from the Bi_2_S_3_ conduction band to the CdS conduction band, enhancing charge separation efficiency while preserving strong redox capability and resulting in significantly improved selectivity for CO_2_ reduction to C_2_H_4_ far beyond single components. Furthermore, this bidirectional exchange strategy has been extended to multidimensional structures. Xu *et al.*^51^ immersed pre-hydrothermally synthesized 2-D Bi_2_WO_6_ nanosheets (BWO) in a TAA solution, the hydrolysis of TAA releases S^2−^ that selectively replaces interlayer O^2−^ in Bi_2_WO_6_, generating Bi_2_S_3_ nanodots *in situ* on the nanosheet surface to form a Bi_2_S_3_/2D-Bi_2_WO_6_ type-II heterojunction. The 2D sheet morphology of Bi_2_WO_6_ is preserved, while Bi_2_S_3_ decoration introduces surface oxygen vacancies and promotes charge separation, effectively lowering charge recombination. Consequently, the composite exhibits a markedly higher visible-light-driven photodegradation rate constant for RhB than pure Bi_2_WO_6_, with sample BWS-2 showing the optimal activity.

Overall, ion exchange enables controlled fabrication of Bi_2_S_3_ heterojunctions at low temperature and ambient pressure. The exchange depth can be tuned continuously from nanodot, thin shell, to core/shell, while the interfacial defect density remains low. These attributes make ion exchange an indispensable post-synthetic tool for constructing high-efficiency photocatalytic systems.

#### 3.1.4. Microwave-assisted method

**Fig. 7:**
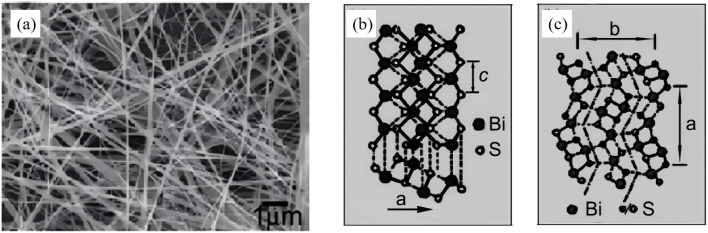
(a) SEM image of the obtained nanowires. (b and c) Schematic structures of the chain-type Bi_2_S_3_.^52^ Copyright 2008, Elsevier.

In recent years, microwave (MW) irradiation has emerged as a promising route for the rapid and energy-efficient fabrication of nanostructured materials. Instantaneous volumetric heating generated by dipolar polarization and ionic conduction enables nucleation and crystallization to be completed within minutes, which conventionally require hours under solvothermal conditions, thereby offering a green alternative for tailoring Bi_2_S_3_ nanoarchitectures. The evolution of MW-assisted Bi_2_S_3_ synthesis can be summarized as a dot -wire-flower sequence, while simultaneously demonstrating the tunability of facet exposure and defect density under the microwave field. As early as 2008, Li *et al.*^52^ reported a one-step MW-solvothermal protocol. In this scenario, Bi(NO_3_)_3_·5H_2_O and Na_2_S·5H_2_O were reacted under MW irradiation, producing single-crystalline nanowires 20 nm in diameter and tens of micrometres in length (yield >95%) (Fig. 7). Compared with conventional solvothermal methods, microwave-assisted heating offers rapid volumetric heating, fast reaction rates, short processing time, high selectivity and energy efficiency, enabling highly efficient anisotropic growth of Bi_2_S_3_ in an extremely short period. This study not only provides a novel route for rapid preparation of Bi_2_S_3_ nanomaterials, but also establishes an important methodological foundation for precise dimensional control of nanomaterials through the tunable microwave power feature. Subsequently, Godzierz *et al.*^53^ employed microwave-assisted synthesis to fabricate flower-like 3D microrods assembled from nanorods. The rapid and uniform heating of the microwave field drastically shortens reaction time while enabling controlled nucleation and growth of Bi_2_S_3_ crystals, effectively regulating the aspect ratio and dispersity of the particles. This result validates the pronounced advantages of the microwave method in tailoring Bi_2_S_3_ morphology and enhancing carrier transport efficiency, which originate from the smaller microrod diameter and higher aspect ratio.

By coupling three operational parameters including power, time and solvent polarity, the MW-assisted approach can accomplish a controllable 1D nanowire and 3D microflower transformation within 10–20 min. Volumetric heating suppresses local overheating and defect aggregation, yielding highly crystalline products with tunable facet exposure. Consequently, microwave synthesis has become a green and scalable alternative for rapidly preparing high-performance Bi_2_S_3_ nanostructures for photocatalytic applications.

#### 3.1.5. Template-directed synthesis

**Fig. 8:**
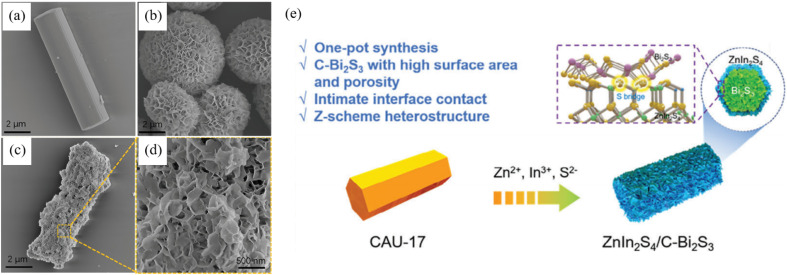
SEM images of (a) CAU-17, (b) ZnIn_2_S_4_ and (c and d) ZCB-1. (e) Creative synthetic method of ZnIn_2_S_4_/Bi_2_S_3_ hybrids.^54^ Copyright 2024, Wiley-VCH Verlag.

The template approach follows a shape-first and sulphidation-second philosophy, in which a removable hard or soft scaffold is erected, and Bi and S precursors are then infiltrated, and ultimately Bi_2_S_3_ is nucleated within the confined space, thereby dictating macro-/meso-scopic morphology and preferential facet exposure. Methodological evolution proceeds through three stages including hard, soft and synergistic process which sequentially solve the challenges of shape replication, interfacial coupling and band alignment. Dai *et al.*^54^ employed rod-like Bi-based MOF CAU-17 as both sacrificial template and Bi source to construct, in one pot, a hierarchical C–Bi_2_S_3_/ZnIn_2_S_4_ heterojunction in which ZnIn_2_S_4_ nanosheets decorate the MOF-derived Bi_2_S_3_ framework (Fig. 8). Benefiting from the inherited CAU-17 architecture, C–Bi_2_S_3_ possesses a high specific surface area of 179 m^2^ g^−1^ (>13 times that of ordinary solvothermal O–Bi_2_S_3_) and abundant mesopores. During the single-step synthesis sulfur acts as a “bridge”, creating intimate inter-layer contact between ZnIn_2_S_4_ and C–Bi_2_S_3_, suppressing nanosheet aggregation and exposing more active sites. Under visible light and ambient air the composite delivers 1178–1324 µmol L^−1^ H_2_O_2_ while degrading >95% of pollutants and markedly lowering chemical oxygen demand (COD). This work first demonstrates that a Bi-MOF hard template can simultaneously tailor morphology and porosity of Bi_2_S_3_-based heterojunctions through structural inheritance and growth confinement, offering a new paradigm for photocatalysts that balance active-site exposure and carrier transport. Mi *et al.*^55^ employed bismuth salicylate (BiSSA) as a hard template and synthesized 1-D Bi_2_S_3_/Bi_4_O_5_Br_2_ S-scheme hierarchical microbundles *via* a simple one-pot solvothermal route. The method achieves uniform dispersion of Bi_2_S_3_ nanoparticles throughout the Bi_4_O_5_Br_2_ matrix, greatly enlarging the heterojunction interface. HRTEM image confirms atomic-scale face-to-face coupling between the Bi_2_S_3_ (211) and Bi_4_O_5_Br_2_ (402) planes. This lattice-matching effect, synergistic with band-structure modulation, accelerates charge separation and migration. Under visible light the microbundles degrade 98% of RhB within 30 min, with a rate constant 3.2 times that of bare Bi_4_O_5_Br_2_ and 137 times that of bare Bi_2_S_3_, far outperforming a conventional surface-supported heterojunction (Ss). The work highlights the unique advantage of hard-template strategies in simultaneously realizing precise facet coupling and band alignment for superior photocatalytic performance.

By combining hard scaffolds for shape confinement and soft interfaces for facet registration, the template route enables low-temperature, ambient-pressure customization of Bi_2_S_3_ thickness, crystal facet and pore structure within a single protocol. The removable nature of the templates eliminates surface-ligand residues and furnishing clean as well as highly active interfaces. Consequently, template-directed synthesis has become a general and powerful strategy for constructing sophisticated Bi_2_S_3_-based heterojunctions tailored to photocatalytic applications.

### 3.2. Morphology–property relationships

#### 3.2.1. Zero-dimensional nanoparticles

**Fig. 9:**
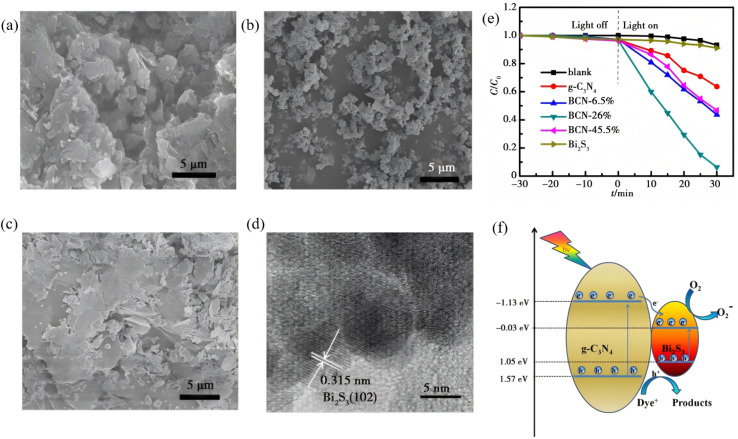
SEM images of (a) pure g-C_3_N_4_, (b) Bi_2_S_3_, (c) BCN-26%. (d) HRTEM images of BCN-26%. (e) Degradation curves of RhB under visible light irradiation using pure g-C_3_N_4_, Bi_2_S_3_, and Bi_2_S_3_/g-C_3_N_4_ heterojunction (f) Schematic illustration of photocatalytic mechanism of Bi_2_S_3_/g-C_3_N_4_ heterojunction under visible light irradiation.^57^ Copyright 2020, Chongqing Southwest Information Co., Ltd.

Zero-dimensional Bi_2_S_3_ nanocrystals (≤10 nm) serve as an ideal model for high-efficiency photocatalysis because of complete exciton confinement, ultra-high surface area and size-tunable band gaps. Establishing their morphology–performance paradigm follows a size–band gap–interface hierarchy. For example, monodisperse quantum dots (QDs) are first obtained *via* mild solution or ultrasonic routes; surface coupling or *in situ* deposition then builds interfacial electric fields; finally, size-dependent carrier separation is quantified. Uddin *et al.*^56^ used soluble starch [(C_6_H_10_O_5_)_*n*_] as both stabilizer and capping agent to synthesize Bi_2_S_3_ nanoparticles (NPs) *via* a one-pot aqueous route. Bi(NO_3_)_3_·5H_2_O and Na_2_S served as Bi and S sources, respectively, and the reaction was conducted stepwise at 70–90 °C. Hydroxyl groups of the starch coordinate with Bi^3+^, which caps the growing nuclei and suppresses aggregation, yielding monodisperse quasi-spherical Bi_2_S_3_ NPs with an average diameter of ∼11 nm (7–15 nm range) and excellent colloidal stability. UV-vis spectroscopy reveals a strong excitonic shoulder at 310 nm, based on which Tauc analysis gives a band gap of 2.86 eV, a pronounced blue-shift relative to bulk Bi_2_S_3_ (1.3 eV), confirming that quantum-confinement-induced discrete energy levels dominate the size-dependent optical properties. Li *et al.*^57^ developed a facile one-step solvothermal route in which Bi(NO_3_)_3_ and Na_2_S_2_O_3_ serve as Bi and S sources, respectively, in ethylene glycol (Fig. 9). Under these conditions, zero-dimensional Bi_2_S_3_ nanoparticles (∼10 nm) are grown *in situ* onto g-C_3_N_4_ nanosheets, yielding a 0D/2D Bi_2_S_3_/g-C_3_N_4_ heterojunction. TEM image confirms the uniform dispersion of the quantum dots across the nanosheet support. Band alignment (g-C_3_N_4_: *E*_g_ = 2.71 eV, CB = −1.13 eV, VB = 1.57 eV; Bi_2_S_3_: *E*_g_ = 1.24 eV, CB = 1.05 eV, VB = −0.33 eV) drives photogenerated electrons from the g-C_3_N_4_ conduction band into the Bi_2_S_3_ conduction band, suppressing charge recombination and prolonging carrier lifetime. The thus-prepared composite demonstrates the extended visible-light absorption, and the 26 wt% Bi_2_S_3_/g-C_3_N_4_ sample exhibits the highest activity, which is remarkably larger than bare g-C_3_N_4_ while retaining stable performance over five cycles. This study not only validates the direct benefit of size-controlled 0D Bi_2_S_3_ quantum dots but also provides a universal framework for designing quantum-dot-based heterojunctions with optimized photocatalytic performance.

#### 3.2.2. One-dimensional nanostructures

**Fig. 10:**
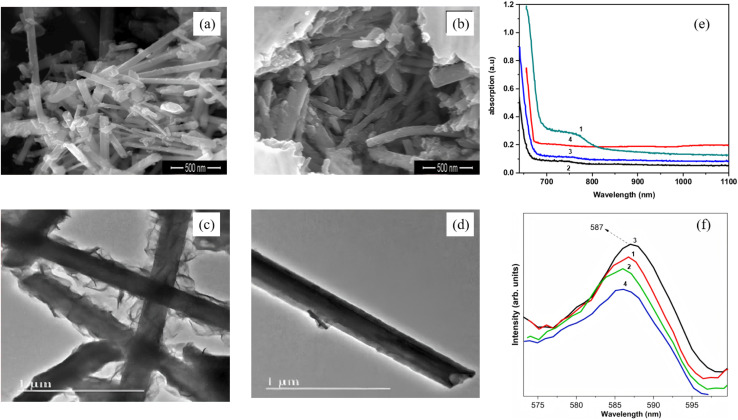
SEM image of Bi_2_S_3_ nanorods prepared with different reaction time (a) 3 h, (b) 4 h. TEM image of Bi_2_S_3_ nanorods prepared with different reaction time (c) 2 h; (d) 3 h. (e) UV-vis spectrum of Bi_2_S_3_ nanorods prepared with different reaction time, (f) Photoluminescence spectra of Bi_2_S_3_ nanorods prepared with different reaction time. (1–4) 1 h, 2 h, 3 h, 4 h.^59^ Copyright 2022, Elsevier BV.

One-dimensional (1-D) Bi_2_S_3_ nanorods/nanowires are regarded as ideal platforms for accelerating photogenerated charge separation because they combine directional charge transport channels, high specific surface area and facet-selective exposure. Establishing their structure–performance paradigm follows a diameter-facet-interface strategy. First, intrinsic extension along [001] is achieved *via* solvothermal or microwave routes. Second, radial size is tuned to optimize band-edge positions. Finally, core–shell or epitaxial heterointerfaces are introduced to realize synergistic photocatalytic and photoelectrochemical enhancement. Liu *et al.*^58^ reported in 2016 a facile two-step hydrothermal-plus-annealing route to grow a novel Bi_2_S_3_-nanowire@TiO_2_-nanorod (Bi_2_S_3_ NWs@TiO_2_ NRs) architecture directly on FTO glass (Fig. 10). Rather than coating Bi_2_S_3_ with TiO_2_, they used a pre-formed TiO_2_ nanorod array as the scaffold and grafted Bi_2_S_3_ nanowires onto its surface. Because the Bi_2_S_3_ conduction-band edge lies above that of TiO_2_, photogenerated electrons are readily injected from Bi_2_S_3_ into TiO_2_ and then conducted along the nanorods to the FTO substrate, while holes remain confined within the Bi_2_S_3_ nanowires, markedly suppressing the charge recombination. Serving as a photoanode, this heterostructure delivers a visible-light-responsive H_2_-evolution rate of 35.97 µmol cm^−2^ h^−1^, outperforming previously reported Bi_2_S_3_/TiO_2_ core–shell nanorods. The work underscores the synergistic benefit of 1D/1D heterostructure for light-to-electricity conversion and offers a surfactant-/template-free route that relies solely on the intrinsic anisotropic growth of Bi_2_S_3_, providing a fresh concept for designing new hetero-nanostructures. Arumugam *et al.*^59^ employed a reflux approach using Bi(NO_3_)_3_ and thiourea as precursors in DMF, with CTAB as surfactant, to controllably synthesize orthorhombic Bi_2_S_3_ nanorods at 180 °C. By tuning reaction time (1–4 h), they established a clear size–morphology–property relationship. After 3 h, uniform 1D rods ∼233 nm in diameter and >1 µm long with optimal crystallinity were obtained, and extending to 4 h caused deformation and shrinkage (diameter down to 140 nm). Optical measurements revealed a pronounced quantum-size effect, that is, the 75 nm rods (1 h) exhibited a 1.91 eV band gap, whereas the 233 nm rods (3 h) showed 1.81 eV. The high-aspect-ratio single-crystal architecture, preferentially grown along (130), enhances carrier mobility and yields high dielectric constant and low loss at low frequency *via* combined interface/orientation polarization. This work not only maps out size-dependent performance rules for 1D Bi_2_S_3_ nanorods but also provides a universal materials-design paradigm for optimizing their use in photocatalysis and photoelectrochemistry.

#### 3.2.3. Two-dimensional nanosheets

**Fig. 11:**
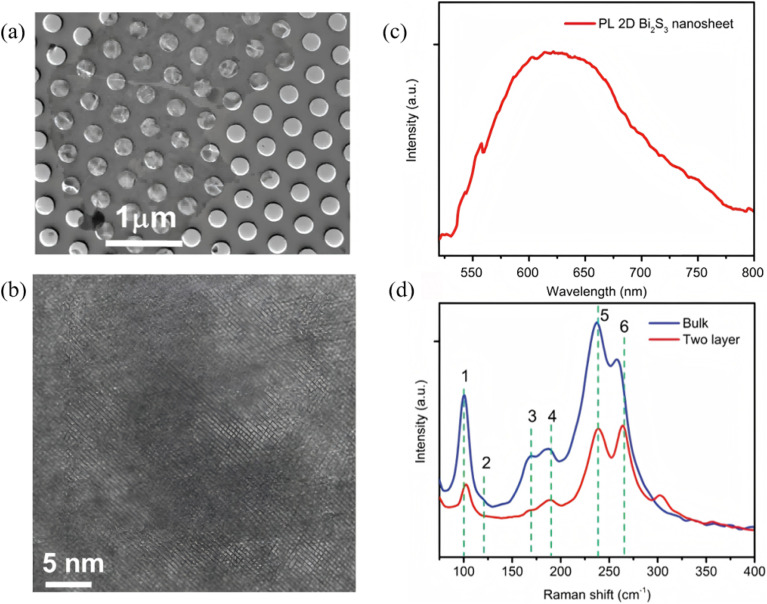
(a) TEM image of Bi_2_S_3_ nanosheet, (b) HRTEM image of Bi_2_S_3_ nanosheet, (c) PL spectrum of a bilayer Bi_2_S_3_ nanosheet, and (d) Raman spectrum of bulk Bi_2_S_3_ (blue line) and 2D nanosheets (red line).^60^ Copyright 2020, John Wiley and Sons Ltd.

**Fig. 12:**
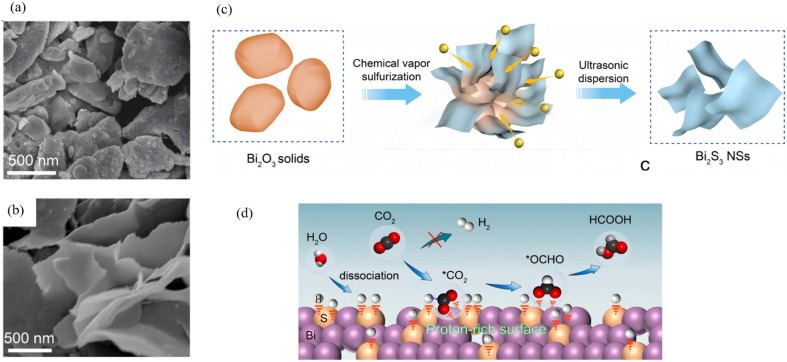
(a and b) SEM images of bulk Bi_2_O_3_ and Bi_2_S_3_ NSs. (c) Schematic illustration of the chemical vapor sulfurization process for the conversion of bulk Bi_2_O_3_ to 2D Bi_2_S_3_ NSs. (d) The proposed reaction mechanism for CO_2_ RR over 2D-Bi with proton-rich surface to stabilize *OCHO for CO_2_ reduction to formate.^61^ Copyright 2024, Elsevier.

Two-dimensional Bi_2_S_3_ nanosheets, which feature atomic thickness, large-area exposure of active facets and tunable in-plane anisotropy, are regarded as an ideal platform for achieving efficient photogenerated charge separation and accelerated surface-reaction kinetics. Single- or few-layer structures are first obtained *via* confined growth or exfoliation, after which defects or lattice strain are introduced to tailor the band-edge positions. Finally, in-plane heterojunctions or defect sites are constructed to realize synergistic enhancements in photocatalytic performance. Messalea *et al.*^60^ developed a liquid-metal-based two-step synthesis method to overcome the difficulty of large-area growth of anisotropic Bi_2_S_3_ crystals. First, molten-bismuth surface-limited oxidation and exfoliation on SiO_2_/Si or glass substrates produced wafer-scale Bi_2_O_3_ nanosheets which were then sulfurized at <450 °C in a tube furnace with elemental sulfur to yield millimetre-scale and high-quality single-crystal Bi_2_S_3_ nanosheets with 1–3 nm thick (typically 2.5 nm) (Fig. 11). The sheets possess an orthorhombic lattice with (210) preferred orientation and pronounced in-plane anisotropy verified by polarized Raman spectroscopy. The few-layer Bi_2_S_3_ is a p-type semiconductor with a direct band gap of ∼2.3 eV and a maximum hole mobility of 28 cm^2^ V^−1^ s^−1^, providing an excellent material and structural platform for high-performance 2D optoelectronics. Li *et al.*^61^ devised a one-step vapor-phase sulfidation with synchronous defect creation protocol wherein high-temperature sulfur vapor reacts with Bi_2_O_3_, while SO_2_ released during the process breaks surface lattice bonds to generate abundant defects, yielding defect-rich 2D Bi_2_S_3_ nanosheets (NSs). These defective NSs undergo dynamic reconstruction into a 2D metallic Bi phase that simultaneously hosts sulfur dopants and lattice defects (Fig. 12). The work demonstrates that cooperative defect engineering and hetero-atom doping can modulate both proton supply and intermediate stabilization, offering a general design paradigm for optimizing 2D Bi-based catalysts.

#### 3.2.4. Three-dimensional hierarchical architectures

**Fig. 13:**
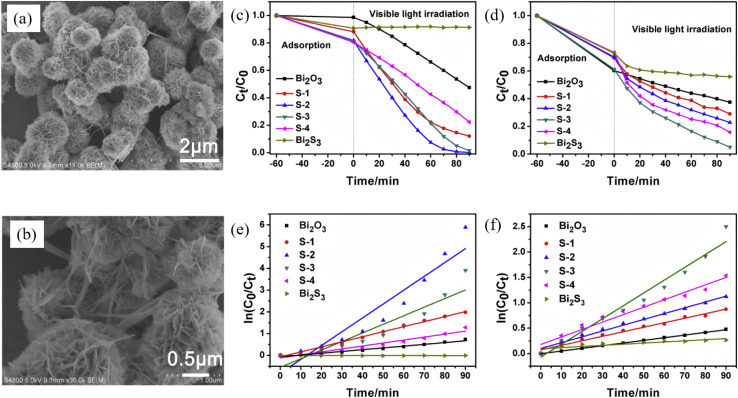
(a and b) SEM images of samples S-3. Photocatalytic removal curves of RhB (c) and Cr(vi) (d). The corresponding kinetics of RhB oxidation (e) and Cr(vi) reduction (f).^45^ Copyright 2019, Copyright 2020, Elsevier.

Three-dimensional Bi_2_S_3_ nanoflowers, self-assembled from 2D nanosheets, combine a high specific surface area, abundantly exposed facets, and hierarchical porosity, simultaneously enhancing light harvesting, mass transfer, and active-site utilization. First, nanosheet self-assembly is achieved *via* a one-step hydrothermal route. Then, *in situ* sulfidation or hetero-epitaxy introduces a built-in electric field, ultimately constructing p–n or Z-scheme architectures that spatially separate oxidation and reduction reactions. Sang *et al.*^45^ employed Bi(NO_3_)_3_·5H_2_O and thiourea as precursors to fabricate 1–2 µm Bi_2_O_3_/Bi_2_S_3_ nanoflowers self-assembled from nanosheets decorated with sparse nanorods *via* a one-step hydrothermal route, generating an inter-sheet mesoporous network (Fig. 13). The intimate contact between p-type Bi_2_S_3_ and n-type Bi_2_O_3_ creates a built-in electric field across the p–n heterojunction. Under visible light, 99.72% of RhB is removed and >91.8% of Cr(vi) is reduced within 90 min, demonstrating that the hierarchical nanoflower simultaneously enhances pollutant adsorption and redox bifunctionality. Photogenerated holes are the main active species for RhB degradation, while photogenerated electrons dominate Cr(vi) reduction. Zhou *et al.*^62^ employed a sol–gel route coupled with *in situ* growth to fabricate Bi_2_S_3_/BiFeO_3_ nanoflower heterojunctions. Using Bi(NO_3_)_3_·5H_2_O and Fe(NO_3_)_3_·9H_2_O as BiFeO_3_ precursors and l-cysteine as the sulfur source, Bi_2_S_3_ was grown directly on the BiFeO_3_ scaffold. The narrow band gap of ferroelectric BiFeO_3_ and the staggered alignment with Bi_2_S_3_ produce a Type-II heterojunction whose built-in field drives spatial charge separation, in which electrons migrate from the Bi_2_S_3_ conduction band to the BiFeO_3_ conduction band, while holes move in the opposite direction, maximizing photocurrent response at a 1 : 3 molar ratio. Under visible light, the composite degrades >99% of malachite green within 2 h (complete removal in 60 min) with optimal activity. The study confirms that the synergism between ferroelectric BiFeO_3_ and Bi_2_S_3_ can amplify the photocatalytic merits of 3D nanostructures, offering a new design concept for high-efficiency Bi_2_S_3_-based photocatalysts in environmental remediation.

## 4. Optimization strategies for Bi_2_S_3_ photocatalysis

### 4.1. Heterojunction construction

#### 4.1.1. Type-II heterojunctions

**Fig. 14:**
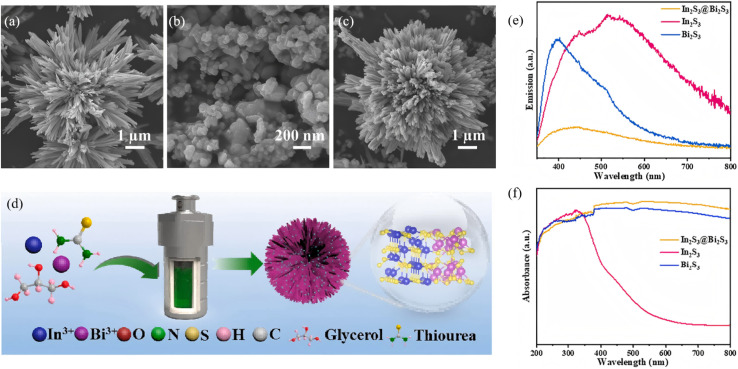
SEM images of (a) Bi_2_S_3_, (b) In_2_S_3_ and (c) In_2_S_3_@Bi_2_S_3_. (d) Schematic illustration of synthesis procedures for In_2_S_3_@Bi_2_S_3_. (e) PL and (f) UV-vis absorption spectra of Bi_2_S_3_, In_2_S_3_ and In_2_S_3_@Bi_2_S_3_.^64^ Copyright 2023, Elsevier.

A Type-II band alignment introduces a staggered offset that spatially separates photogenerated electrons and holes, prolonging carrier lifetime and suppressing charge recombination. Owing to its narrow gap (∼1.3 eV) and large absorption coefficient, Bi_2_S_3_ can act as either electron donor or acceptor in Type-II assemblies. Lian *et al.*^63^ employed BiVO_4_ nanorods as a core and carried out *in situ* surface sulfidation to co-grow a Bi_2_S_3_ shell. In this nanostructure, the two phases share Bi atoms, mutually coupling their electronic structures and reducing interfacial lattice mismatch. *In situ* impedance spectroscopy revealed a carrier-transport activation energy (CTAE) as low as 0.261 eV, markedly lowering the energy barrier for charge separation and migration. Benefiting from atomic-scale interfacial contact, enhanced visible-light absorption (edge extended to 1007 nm) and highly efficient carrier separation, BiVO_4_@Bi_2_S_3_ exhibits dramatically improved photocatalytic Cr(vi) reduction with 50 ppm Cr(vi) completely reduced within 40 min, along with an apparent rate constant 35.5 times that of pure BiVO_4_. This work first demonstrates that sharing atoms to build an inorganic heterojunction can effectively weaken coulombic repulsion between the two phases, offering a new strategy for boosting carrier-migration efficiency in Type-II heterojunctions. Dang *et al.*^64^ fabricated In_2_S_3_@Bi_2_S_3_ core–shell nanoflowers *via* a one-pot hydrothermal route (Fig. 14). Leveraging narrow band gap and high visible-light absorption of Bi_2_S_3_ together with strong photosensitivity and photocorrosion resistance of In_2_S_3_, the pair form a type-II heterojunction that spatially separates photogenerated carriers. During discharge, electrons from the In_2_S_3_ conduction band migrate to Bi_2_S_3_ to drive oxygen-reduction, while holes from the Bi_2_S_3_ valence band move to In_2_S_3_ to oxidize discharge products. Light energy deepens the reaction depth and accelerates charge exchange, delivering bifunctional catalysis. This strategy couples visible-light harvesting with electrochemical energy storage through Type-II band engineering, offering a new avenue for addressing energy shortages *via* photo-assisted Li–O_2_ batteries. Yuan *et al.*^65^ fabricated a Bi-TCPP/Bi_2_S_3_ heterojunction *via* a one-pot route. Interaction between the metalloporphyrin Bi-TCPP and Bi_2_S_3_ generates oxygen vacancies (Ov) that prolong carrier lifetime, enhance light absorption and activate reactants. A Type-II band alignment drives electrons from Bi_2_S_3_ to Bi-TCPP and holes in the opposite direction, suppressing charge recombination. For the first time the “Ov + Type-II” strategy was applied to photocatalytic Cr(vi) reduction, extending Type-II heterojunctions to environmental remediation.

Collectively, these studies push Type-II interfacial charge-transfer efficiency to new levels while achieving full visible-to-near-infrared absorption and spatial separation of oxidative and reductive sites, offering a universal design framework for high-performance Bi_2_S_3_-based full-spectrum photocatalytic systems.

#### 4.1.2. Z-scheme heterojunctions

**Fig. 15:**
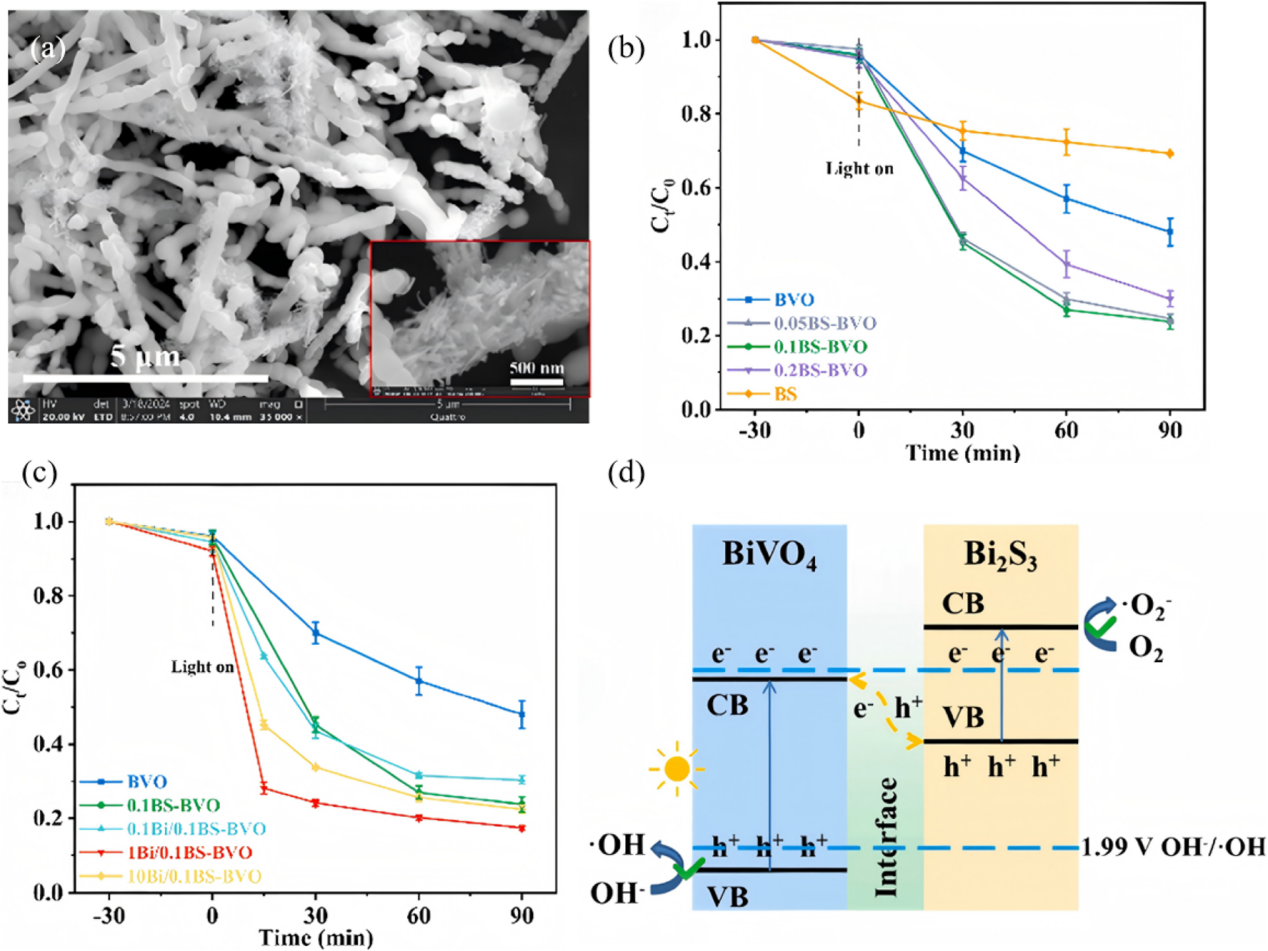
(a) SEM images of 1Bi/0.1BS-BVO nano-chains. Photodegradation performance of TCH with (b) BS-BVO, and (c) Bi/BS-BVO. (d) Schematic diagram of the band structure: Z-scheme heterojunction of BS-BVO.^66^ Copyright 2025, Elsevier.

Z-scheme architectures preserve the strongest oxidation and reduction potentials of each semiconductor by forcing photogenerated electrons and holes to recombine across the junction, and are therefore considered the ultimate platform for full-spectrum photocatalysis. Thanks to its narrow band gap and deep-lying valence band, Bi_2_S_3_ frequently serves as the visible-to-NIR-responsive electron donor in such systems. Fan *et al.*^66^ fabricated a 1D Bi/Bi_2_S_3_–BiVO_4_ Z-scheme composite that delivers outstanding performance for tetracycline degradation (Fig. 15). Metallic Bi broadens the light-harvesting window *via* localized surface plasmon resonance (LSPR), suppresses carrier recombination, and participates in a tri-phase heterojunction to accelerate charge transfer. Bead-like BiVO_4_ decorated with needle-like Bi_2_S_3_ increases the specific surface area and shortens carrier-transport paths. The system simultaneously preserves the strongly oxidative holes of the BiVO_4_ valence band (VB) and the highly reductive electrons of the Bi_2_S_3_ conduction band (CB), raising the tetracycline degradation rate by more than 3 times compared with pure BiVO_4_. Recently, Guo *et al.*^67^ reported a Z-scheme Bi_2_S_3_/Ag_2_S heterojunction that harvests photons across the entire UV-to-NIR window. Within the junction, electrons in the Ag_2_S conduction band recombine with holes in the Bi_2_S_3_ valence band, leaving Ag_2_S CB electrons with strong reducing power and Bi_2_S_3_ VB holes with strong oxidizing power. The optimized Bi_2_S_3_/Ag_2_S sample delivers high degradation efficiencies under all spectral regions, that is, 86% under UV (8 times that of bare Bi_2_S_3_), 84% under visible (5 times), and 88% under NIR (4.4 times) while retaining stable performance after five consecutive cycles. This extends Z-scheme photocatalysis into the near-infrared region, surpassing the limitations of single-phase NIR photocatalysts. The outstanding organic-pollutant purification is directly ascribed to the staggered band alignment and the internal electric field directed from Ag_2_S to Bi_2_S_3_, which preserves the coexistence of strongly reductive Ag_2_S CB electrons and highly oxidative Bi_2_S_3_ VB holes.

Building on this, Chachvalvutikul *et al.*^68^ fabricated a direct Z-scheme Bi_2_S_3_/ZnIn_2_S_4_ photocatalyst that exhibits markedly enhanced activity toward methylene-blue degradation. Photogenerated electrons in the Bi_2_S_3_ CB jump to the ZnIn_2_S_4_ VB and recombine there, leaving strongly reducing electrons in the ZnIn_2_S_4_ CB (−0.97 eV *vs.* NHE) to reduce O_2_ to ˙O_2_^−^, and strongly oxidizing holes in the Bi_2_S_3_ VB to attack MB directly. The ˙O_2_^−^ radicals are further reduced to ˙OH radicals that assist in dye decomposition. The 12.5 wt% Bi_2_S_3_/ZnIn_2_S_4_ composite shows the best performance, achieving 95.4% MB removal in 300 min. It is far superior to bare ZnIn_2_S_4_ (64.2%) and still retains 87.4% efficiency after three cycles, confirming excellent stability. This fully validates the unique advantage of the Z-scheme charge-transfer pathway in simultaneously preserving the powerful reducing electrons of ZnIn_2_S_4_ and the strong oxidizing holes of Bi_2_S_3_.

#### 4.1.3. p–n junctions

**Fig. 16:**
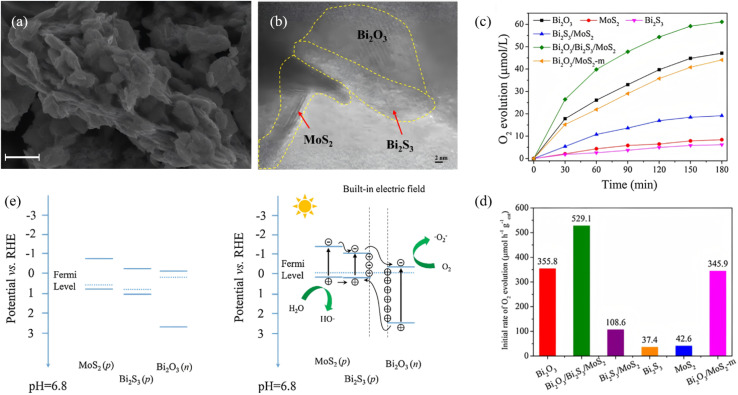
(a) SEM images of Bi_2_O_3_/Bi_2_S_3_/MoS_2_. (b) High-resolution TEM images of Bi_2_O_3_/Bi_2_S_3_/MoS_2_. (c) Photocatalytic activity for O_2_ evolution. (d) Initial water oxidation rate of the different samples under simulated solar light irradiation. (e) Schematic diagram for energy band of Bi_2_O_3_, MoS_2_, and Bi_2_S_3_ and the formation of the three-phase p–n heterojunction and the possible charge separation.^69^ Copyright 2017, Elsevier.

A p–n junction separates photogenerated electrons and holes through a built-in electric field (BEF), providing a classical yet powerful route to enhance Bi_2_S_3_ photocatalysis. Recent studies have quantitatively correlated junction-field strength, depletion width and resulting photoresponse. Sang *et al.*^45^ produced self-assembled Bi_2_O_3_/Bi_2_S_3_ nanoflowers through a one-pot hydrothermal process, forming an intimate native p–n junction that drives efficient charge separation for enhanced photocatalytic performance. The built-in electric field drives electrons toward n-type Bi_2_O_3_ while holes remain in p-type Bi_2_S_3_, furnishing a strong force for carrier separation. Thus, 99.72% of RhB is degraded and Cr(vi) is reduced within 90 min. Ke *et al.*^69^ constructed a n-Bi_2_O_3_/p-Bi_2_S_3_/p-MoS_2_ triple p–n heterojunction by inserting a p-type Bi_2_S_3_ interlayer between n-type Bi_2_O_3_ and p-type MoS_2_ (Fig. 16). The engineered Fermi-level gradient suppresses electron–hole recombination and accelerates charge separation/transport. The resulting Bi_2_O_3_/Bi_2_S_3_/MoS_2_ delivers a water-oxidation rate of 529.1 µmol h^−1^ g^−1^ that is 1.5 times that of bare Bi_2_O_3_ and 12.5 times that of bare MoS_2_. At the same time, it achieves 90% MB removal in 6 h. The Bi_2_S_3_ interlayer ensures favorable p–n band alignment, and the high conductivity of MoS_2_ further boosts charge transfer, synergistically enhancing overall photocatalytic activity. Latifian *et al.*^70^ introduced 0.3%, 0.6% and 1% Ti^4+^ into n-Bi_2_S_3_ (optimum 0.6%) to fabricate a Ti-doped Bi_2_S_3_/NiO p–n heterojunction. The study revealed that Ti incorporation not only tailors the band structure of Bi_2_S_3_ but also enables highly efficient separation of photogenerated carriers *via* the built-in electric field of the p–n junction with NiO. Under visible-light irradiation the optimal catalyst (0.6% Ti–Bi_2_S_3_/1% NiO) degrades 80% of MB within 500 min, demonstrating markedly enhanced photocatalytic activity.

#### 4.1.4. Schottky junction

**Fig. 17:**
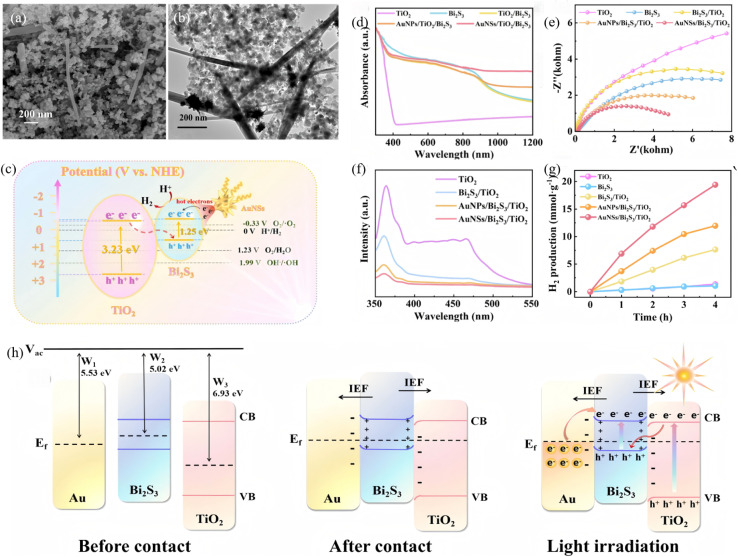
(a) SEM and (b) TEM images of Au NSs/Bi_2_S_3_/TiO_2._ (c) Schematic representation of Schottky/S-scheme charge transfer mechanisms. (d) DRS spectra, (e) Electrochemical impedance spectroscopy, (f) PL spectra, (g) H_2_ evolution performances of different samples, (h) Schottky/S-scheme heterojunction mechanisms including before contact, after contact, and under light irradiation.^72^ Copyright 2025, Royal Society of Chemistry.

A Schottky junction creates an ultrafast electron-extraction pathway across the metal–semiconductor interface, effectively suppressing the surface charge recombination and prolonging hole lifetime in Bi_2_S_3_. Recent efforts have quantitatively linked Schottky-barrier height, charge-transfer kinetics and resulting photocatalytic activity. Hosseini *et al.*^71^ first converted Ti_3_C_2_ MXene into a 3D TiO_2_@Ti_3_C_2_ framework by hydrothermal oxidation, then electrostatically assembled 2D Bi_2_S_3_ nanosheets onto the surface to obtain a hierarchical TiO_2_@Ti_3_C_2_/Bi_2_S_3_ Schottky–Z-scheme junction. Ti_3_C_2_ MXene serves as an interlayer that interfaces simultaneously with TiO_2_ and Bi_2_S_3_, greatly accelerating charge separation. The synergistic Schottky–Z-scheme synergy makes Ti_3_C_2_ act as a Schottky barrier that speeds photo-carrier extraction while the Z-pathway preserves the strong redox potentials of both semiconductors, suppressing electron–hole recombination. Under visible light, TiO_2_@Ti_3_C_2_/20% Bi_2_S_3_ degrades 84.13% of 40 mg L^−1^ tetracycline in 135 min with a rate constant 3 times that of TiO_2_@Ti_3_C_2_. The ˙O_2_^−^ and ˙OH radicals are determined as the dominant species. TiO_2_@Ti_3_C_2_/1% Bi_2_S_3_ delivers the highest H_2_-evolution rate (14 141.23 µmol g^−1^ h^−1^) that is 2.37 times that of TiO_2_@Ti_3_C_2_. Both optimal catalysts retain activity and crystal integrity after three cycles, demonstrating excellent recyclability. Sun *et al.*^72^ constructed an Au NSs/Bi_2_S_3_/TiO_2_ double-heterojunction, wherein a Schottky contact between Au NSs and Bi_2_S_3_ and an S-scheme junction between Bi_2_S_3_ and TiO_2_ is formed (Fig. 17). The surface-plasmon resonance (SPR) of Au NSs pushes absorption into the near-infrared (NIR) region, and the narrow-band-gap Bi_2_S_3_ (∼1.3 eV) covers the visible region, while TiO_2_ responds to UV, collectively achieving full UV-Vis-NIR spectral coverage. Under NIR excitation, hot electrons generated in Au NSs are injected into the Bi_2_S_3_ CB *via* the Schottky junction. The H_2_-evolution rate of Au NSs/Bi_2_S_3_/TiO_2_ reaches 5.754 mmol g^−1^ h^−1^, markedly higher than that of either single junction. TRPL reveals a shortened carrier lifetime (0.23 ns *vs.* 0.79 ns for pristine TiO_2_), confirming that the dual junction accelerates charge separation and transfer. This synergistic double-junction design simultaneously broadens the spectral window and enhances redox power.

### 4.2. Element doping

#### 4.2.1. Metal-ion doping

**Fig. 18:**
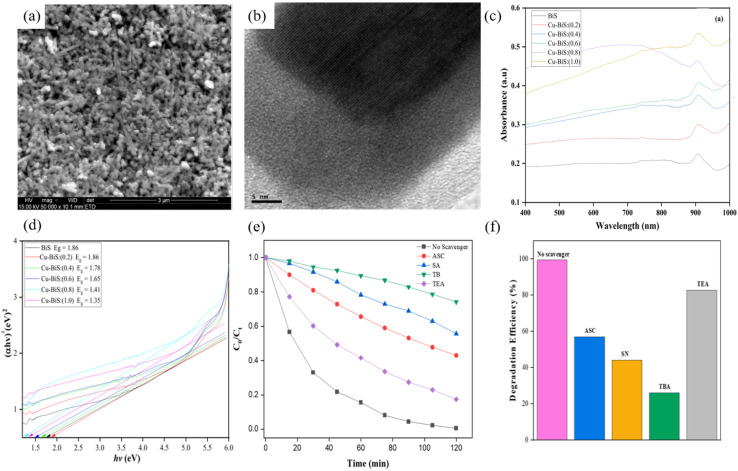
(a) SEM and (b) HRTEM images of Cu–BiS (0.4). (c and d) UV-vis-NIR absorption spectra of (c) pure Bi_2_S_3_ and doped Bi_2_S_3_ nanoparticles with different dopant concentration (0.2, 0.4, 0.6, 0.8 and 1.0 moL) with corresponding (d) Tauc plots. (e and f) UV-visible absorption spectra of (e) degradation percentage of MO with change in time; and (f) the corresponding photodegradation efficiency (ASC, SA, TB, and TEA).^74^ Copyright 2023, Elsevier BV.

**Fig. 19:**
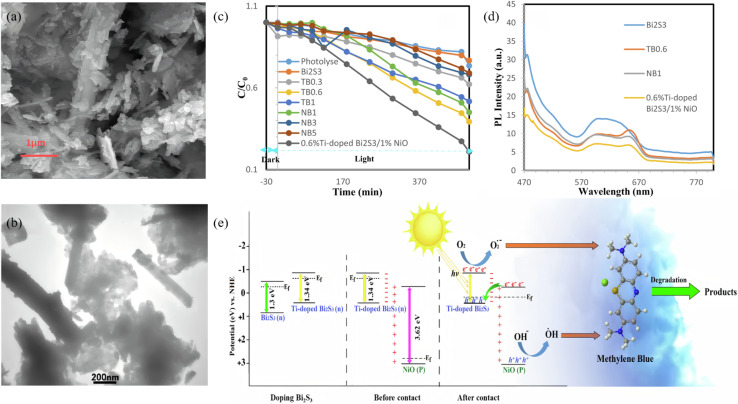
(a) SEM image of 0.6% Ti-doped Bi_2_S_3_/1% NiO and (b) TEM image of 0.6% Ti-doped Bi_2_S_3_/1% NiO. (c) Photodegradation plots of MB over different samples. (d) PL spectra of Bi_2_S_3_, TB0.6, NB1, and 0.6% Ti-doped Bi_2_S_3_/1% NiO. (e) The schematic diagrams of formation of Ti doping and p–n heterojunction and the photocatalytic degradation mechanism of MB by 0.6% Ti-doped Bi_2_S_3_/1% NiO.^70^ Copyright 2023, Elsevier.

Substitutional metal ions introduce extra carriers and mid-gap states into the Bi_2_S_3_ lattice, systematically enhancing visible-light harvesting, carrier lifetime and surface reaction kinetics. Zhou *et al.*^73^ employed a two-step protocol, *i.e.*, SILAR followed by hydrothermal treatment to fabricate Ho^3+^-doped Bi_2_S_3_ thin films. Incorporation of Ho introduces an impurity level that narrows the band gap from 1.67 eV to 1.50 eV, lowering the electron transition energy and increasing the number of photogenerated carriers. As a result, the Ho^3+^-modified film exhibits a 30% higher photovoltage, a 1.97 times larger photocurrent density, and a three-order-of-magnitude increase in carrier concentration, confirming that Ho doping significantly improves the photoelectronic properties of Bi_2_S_3_. Nkwe *et al.*^74^ prepared Cu^2+^-doped Bi_2_S_3_ nanorods *via* a solvothermal route (Fig. 18). Cu^2+^ substitutes for Bi^3+^, which is beneficial for injecting extra electrons into the lattice. This substitution broadens visible-light absorption, tailors the local electron density to optimize the band structure, and markedly enhances surface reactivity. The prolonged carrier lifetime increases the photocatalytic degradation rate constant for methyl orange (MO) by 1.5 times compared with undoped Bi_2_S_3_. Du *et al.*^75^ hydrothermally coupled Cu-doped Bi_2_S_3_ with BiOCl to form an n–n BiOCl/Cu-doped Bi_2_S_3_ heterojunction. Wherein Cu^2+^ acts as an interfacial mediator that traps photoelectrons from the BiOCl CB, preventing electron–hole recombination. These captured electrons subsequently reduce O_2_ to ˙O_2_^−^ radicals, boosting oxidation power. The heterojunction accelerates charge transfer and enlarges the specific surface area to 40.54 m^2^ g^−1^ (*vs.* 23.70 m^2^ g^−1^ for BiOCl and 6.78 m^2^ g^−1^ for Cu-doped Bi_2_S_3_), increasing reactant adsorption and the number of active sites. The composite achieves 97.1% degradation of ciprofloxacin (CIP) within 20 min, far exceeding undoped/un-coupled systems (*i.e.*, Cu-doped Bi_2_S_3_ 5%, bare Bi_2_S_3_ 13%, and BiOCl 67%), exemplifying the doping and heterojunction synergy that amplifies interfacial charge separation. Latifian *et al.*^70^ deposited NiO onto Ti^4+^-doped Bi_2_S_3_ to form a Ti–Bi_2_S_3_/NiO p–n heterojunction (Fig. 19). The markedly enhanced visible-light-responsive photocatalytic activity provides direct evidence that metal-ion doping plays a pivotal role in complex band engineering, in which Ti^4+^ down-shifts the CB minimum, while the p-type NiO possesses a higher VB edge. The resulting graded energetics strengthen the built-in electric field, promoting separation of photogenerated electron–hole pairs. Under visible light, the MB degradation rate is 1.76 times that of pristine Bi_2_S_3_, demonstrating that multi-element co-doping can simultaneously amplify both the band gradient and the internal electric field.

#### 4.2.2. Non-metal ion doping

**Fig. 20:**
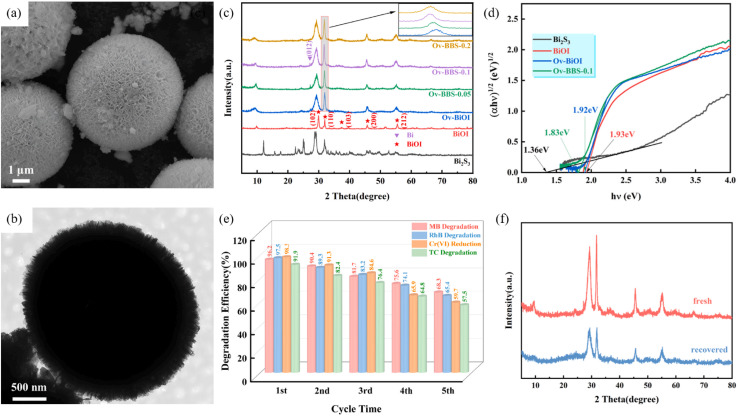
(a) SEM and (b) TEM images of Ov-BBS-0.1 (c) XRD patterns of Bi_2_S_3_, BiOI, Ov-BiOI, and Ov-BBS composites. (d) Curves of (*αhv*)^1/2^*versus hv* of Bi_2_S_3_, BiOI, Ov-BiOI and Ov-BBS-0.1. (e) Cycling tests for the photocatalytic removal of MB, RhB, Cr(vi) and TC by Ov-BBS-0.1 catalyst. (f) XRD pattern of the fresh and recovered Ov-BBS- 0.1 catalyst.^76^ Copyright 2024, ELSEVIER SCI LTD.

Non-metal ions can hybridise valence orbitals, passivate defects and induce band-edge shifts in the Bi_2_S_3_ framework, delivering simultaneous enhancement of visible-to-NIR response and carrier lifetime. Shi *et al.*^76^ fabricated a 3D hierarchical porous BiOI–Bi_2_S_3_ S-scheme heterojunction (Ov-BBS) (Fig. 20) *via* a one-step solvothermal route in which sulfur doping was used to tune the oxygen-vacancy content. After sulfur introduction the absorption edge red-shifted and the band gap of Ov-BBS-0.1 narrowed to 1.83 eV (*vs.* 1.93 eV for bare BiOI). DFT calculations and photoelectrochemical tests confirmed that sulfur doping and oxygen vacancies cooperate to optimize carrier separation and suppress charge recombination. Under UV-vis illumination, Ov-BBS-0.1 efficiently degrades/reduces four typical pollutants including MB, RhB, Cr(vi) and Tetracycline (TC). This work delivers an efficient, stable and broadly applicable Ov-BBS photocatalyst and offers a new strategy for designing high-performance photocatalysts for wastewater treatment.

### 4.3. Surface modification and defect engineering

#### 4.3.1. Surface passivation

**Fig. 21:**
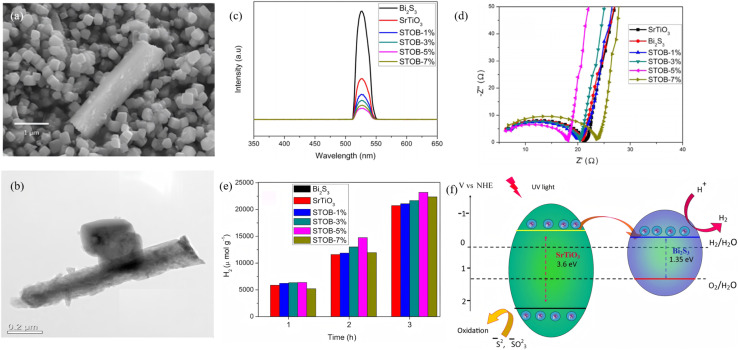
(a) SEM and (b) TEM images of SrTiO_3_/Bi_2_S_3_ (5%) heterojunction. (c) PL, (d) EIS analysis, and (e) photocatalytic hydrogen production performances of SrTiO_3_, Bi_2_S_3_ and SrTiO_3_/Bi_2_S_3_ heterojunction. (f) Possible photocatalytic mechanism of the SrTiO_3_/Bi_2_S_3_ heterojunction.^77^ Copyright 2021, American Chemical Society.

Surface defects are the dominant recombination centers for Bi_2_S_3_ photogenerated carriers. Passivation strategies therefore aim to lower surface-state density, introduce protective overlayers or create space-charge screening, thereby simultaneously extending carrier lifetime and boosting photocatalytic activity. Ganapathy *et al.*^77^ prepared SrTiO_3_ nanocubes and Bi_2_S_3_ nanorods *via* hydrothermal and microwave-assisted methods, respectively, then mixed them *via* ultrasonic dispersion, stirring, centrifugation and drying to obtain heterojunctions denoted STOB-1%, 3%, 5% and 7% (Fig. 21). Time-resolved photoluminescence (TRPL) revealed the fluorescence lifetime of bare SrTiO_3_ and STOB-5%. Acting as a wide-band-gap passivation layer, SrTiO_3_ creates an electron-reflecting barrier that blocks surface-defect trapping in Bi_2_S_3_, reducing charge recombination and quenching the PL peak, which indirectly evidences that passivation lowers surface-state density. When the Bi_2_S_3_ content exceeds 5% (*e.g.*, STOB-7%), the PL intensity recovers. The authors attribute this to increased Bi_2_S_3_ surface-related defects, which enhances carrier recombination and slightly lower the H_2_-evolution rate (STOB-7%: 7.4 mmol g^−1^ < STOB-5%: 7.7 mmol g^−1^). Vu *et al.*^78^ developed a one-step hydrothermal route that uses thiourea as the sulfur source and finely tunes the reaction kinetics of Bi^3+^ and Mo^6+^ to grow ultrathin MoS_2_ flakes directly on Bi_2_S_3_ nanorods, yielding a hierarchical Bi_2_S_3_@MoS_2_ heterojunction. Because the solubility product of Bi_2_S_3_ (1.0 × 10^−97^) is orders of magnitude smaller than that of MoS_2_ (2.2 × 10^−56^), Bi^3+^ reacts first with the sulfur source to form Bi_2_S_3_ nanorods; once Bi^3+^ is depleted. Mo^6+^ is reduced *in situ* to Mo^4+^ by thiourea and nucleates on the Bi_2_S_3_ surface, producing a conformal coating of MoS_2_ nanosheets. The resultant heterostructure, with well-matched energy levels, promotes efficient separation of photogenerated carriers, achieving a total RhB removal of 97.5% and retaining stable performance after three consecutive cycles. Zha *et al.*^79^ modulated oxygen vacancies (OVs) and interfacial chemistry to suppress charge recombination and stabilize active sites, thereby boosting photocatalytic NO oxidation. Annealing the BiVO_4_–Bi_2_S_3_ heterojunction in N_2_ at 340 °C for 5 h generated Ovs that introduce defect levels which act as electron traps, capturing photogenerated electrons and inhibiting e^−^–h^+^ recombination. A subsequent O_2_ atmosphere “repair” at 480 °C for 4 h refilled the Ovs, demonstrating the reversible nature of the passivation. After Ovs are healed, carrier-transport efficiency drops and the photocurrent decreases from 6.4 µA cm^−2^ (OV sample) to 1.95 µA cm^−2^. The Ov-BiVO_4_–Bi_2_S_3_ achieves 63.2% NO removal, 1.8 times higher than the untreated BiVO_4_–Bi_2_S_3_ control (35.2%). After 20 cycles, the ESR signal of Ov-BiVO_4_–Bi_2_S_3_ shows no obvious decay, confirming that the Ovs remain stable and the passivation effect is durable. By way of this post-passivation strategy, the dominant product is NO_3_^−^ with negligible NO_2_^−^, minimizing secondary pollution. Collectively, these studies reduce Bi_2_S_3_ surface defect density by more than one order of magnitude and establish a direct correlation among defect density, carrier lifetime and photocatalytic activity, furnishing a universal passivation framework for high-efficiency Bi_2_S_3_-based photocatalytic systems.

#### 4.3.2. Engineering sulfur-vacancy-rich Bi_2_S_3_

**Fig. 22:**
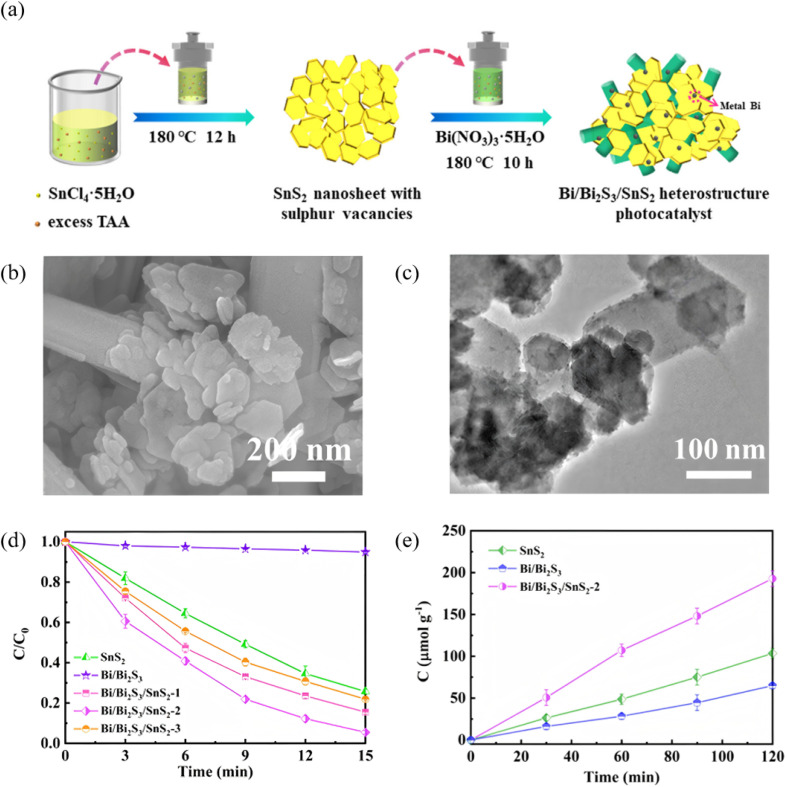
(a) Diagram for the synthetic process of Bi/Bi_2_S_3_/SnS_2_ heterostructure. (b) SEM and (c) TEM images of Bi/Bi_2_S_3_/SnS_2_-2. (d) Reduction curves of Cr(vi) by SnS_2_, Bi/Bi_2_S_3_, and Bi/Bi_2_S_3_/SnS_2_. (e) Nitrogen fixation performance of SnS_2_, Bi/Bi_2_S_3_, and Bi/Bi_2_S_3_/SnS_2_-2.^80^ Copyright 2023, Chinese Society of Metals.

Sulfur vacancies (Sv) introduce mid-gap states and activate surface Bi sites, simultaneously enhancing visible-to-NIR absorption, prolonging carrier lifetime and tailoring reaction selectivity. Lan *et al.*^80^ used SnCl_4_·5H_2_O as the tin source and deliberately added excess thioacetamide (TAA) to synthesize SnS_2_ nanosheets rich in sulfur vacancies (Sv) *via* a hydrothermal route. The surplus sulfur in TAA is essential for vacancy formation. Ethylene glycol then served as both solvent and mild reductant for *in situ* deposition of Bi/Bi_2_S_3_ on the defective SnS_2_ (Fig. 22). Samples containing Sv (SnS_2_-*x* and Bi/Bi_2_S_3_/SnS_2_-*x* series) exhibited markedly higher Cr(vi)-reduction and nitrogen-fixation activities than Sv-free SnS_2_-0. Electron paramagnetic resonance (EPR) showed that the Bi/Bi_2_S_3_/SnS_2_-2 heterostructure possessed the strongest Sv signal, evidencing the highest vacancy concentration. DFT calculations revealed that these high-density Sv sites act as catalytically active centers that accelerate N_2_ adsorption/activation, giving the most favorable N_2_ adsorption energy. Moreover, the enriched Sv sites narrow the SnS_2_ band gap, extend visible-light absorption and thus enhance the overall photoresponse. Further work^81^ also demonstrated that a one-step route to create a sulfur-vacancy-rich SnS/Bi_2_S_3_ Z-scheme photocatalyst yields intimate interfacial contact between SnS and Bi_2_S_3_. The resulting built-in electric field drives carriers along a Z-type pathway, effectively suppressing electron–hole recombination. The abundant sulfur vacancies additionally enhance charge separation and accelerate electron transfer, endowing the heterojunction with both high carrier separation efficiency and strong surface reaction activity. Zhu *et al.*^82^ successfully synthesized a Bi_2_S_3_ catalyst co-optimized by dendritic ultrathin nanosheets and sulfur vacancies. Among the samples, Vs-BS1 (annealed for 1 h) exhibited the best performance. The ultrathin architecture enhances charge separation efficiency and specific surface area, while sulfur vacancies boost photothermal conversion, stabilize reaction intermediates, and lower the thermodynamic energy barrier. The synergistic effect achieved a CO yield of 250 µmol g^−1^ h^−1^*via* photothermal CO_2_ reduction, which is five times higher than that of pure photocatalysis and 3.5 times that of pristine Bi_2_S_3_. This study provides a new avenue for optimizing photothermal catalytic materials through structure design and defect engineering and offers a highly efficient candidate catalyst for CO_2_ conversion and utilization. Collectively, these works establish sulfur-vacancy engineering as a pivotal tool for optimizing Bi_2_S_3_-based catalysis and provide a transferable roadmap for precise vacancy control in energy and environmental applications.

### 4.4. Co-catalyst loading

#### 4.4.1. Noble-metal co-catalysts

**Fig. 23:**
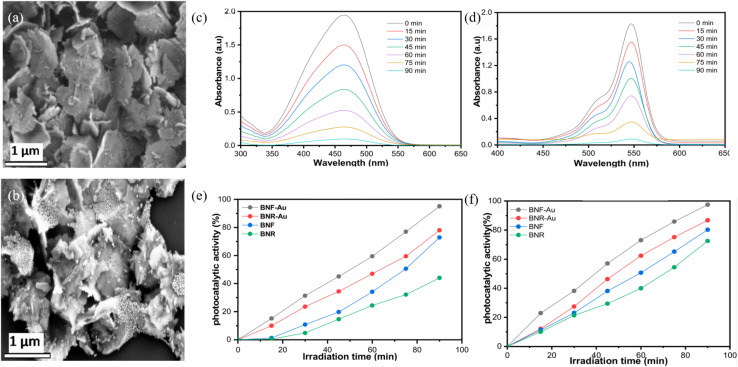
(a) SEM images of flower-shaped Bi_2_S_3_ nanocrystals (BNF) and (b) BNF–Au. Representative absorbance changes of (c) MO, (d) RhB using BNR-Au, and the percentages degradation efficiency by the nanocrystals and corresponding gold decorated analogues for (e) RhB and (f) MO.^83^ Copyright 2021, MDPI.

Noble metals exhibit tunable Fermi levels, strong LSPR effect and high electron-storage capacity, enabling the formation of Schottky junctions or electron reservoirs on Bi_2_S_3_ that markedly accelerate charge separation and surface reaction kinetics. Nwaji *et al.*^83^ developed a simple and low-cost route to prepare Au-nanoparticle-decorated Bi_2_S_3_ heterostructure photocatalysts (Fig. 23). A one-pot colloidal wet-chemistry protocol deposited Au NPs (∼15 nm) onto Bi_2_S_3_ nanorods and nanoflowers, markedly boosting wastewater-treatment performance. The LSPR effect of Au NPs intensifies light absorption and accelerates charge separation, raising degradation efficiencies for MO and RhB to 97.4% and 95.1%, respectively, which 1.2 to 3 times higher than bare Bi_2_S_3_. ˙OH and ˙O_2_^−^ are determined as the dominant active species, and the material retains high stability and recyclability under simulated sunlight irradiation, offering a new design concept for low-cost and high-performance photocatalysts and highlighting the dual plasmonic catalytic role of noble-metal cocatalysts in full-spectrum environmental remediation. These studies establish that noble-metal decoration not only suppresses electron–hole recombination but also supplies abundant active sites and plasmonic heat, providing a versatile blueprint for the design of high-performance Bi_2_S_3_ photocatalysts.

#### 4.4.2. Non-noble-metal cocatalysts

**Fig. 24:**
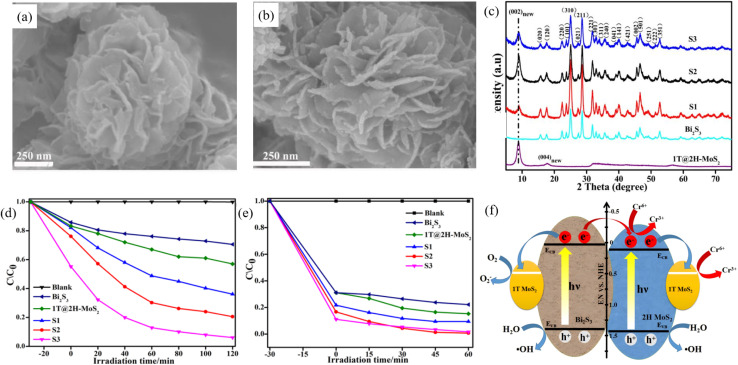
SEM image of (a) 1T@2H-MoS_2_, and (b) Bi_2_S_3_/1T@2H-MoS_2_. (c) XRD pattern from 1T@2H-MoS_2_, Bi_2_S_3_, and Bi_2_S_3_/1T@2H-MoS_2_ heterojunctions with different molar ratios of 1T@2H-MoS_2_. (d and e)Photo-reduction rates of potassium dichromate (d) and MB (e) under visible irradiation. (f) Schematic illustration of the photo-charge separation process over Bi_2_S_3_/1T@2H-MoS_2_.^84^ Copyright 2019, Elsevier.

Earth-abundant alternatives offer low cost and high activity, forming junctions on Bi_2_S_3_ that enables bidirectional electron/hole shunting. Liu *et al.*^84^ fabricated a Bi_2_S_3_/1T@2H-MoS_2_ composite *via* a microwave-hydrothermal route in which Bi_2_S_3_ sheathes the edges of 1T@2H-MoS_2_ (Fig. 24). The 1T phase accounts for 70.2%. The hybrid MoS_2_ delivers dual-face synergy whereby the 2H phase acts as a photosensitizer and the metallic 1T phase accelerates charge transfer. Bare Bi_2_S_3_ and 1T@2H-MoS_2_ reduce only 18% and 32% of Cr(vi), respectively, whereas sample S2 (1 : 1 molar ratio) achieves 89% reduction far superior to other ratios. S2 also degrades 96% of MB *versus* 28% for pure Bi_2_S_3_ and 70% for 1T@2H-MoS_2_ alone. Leveraging the photosensitizer-cocatalyst dual role of 1T@2H-MoS_2_ and the cooperative band alignment, the composite broadens light absorption and uses the metallic conductivity of 1T-MoS_2_ to speed interfacial charge transfer, and thus accepts electrons from 2H-MoS_2_, markedly suppressing electron–hole recombination and outperforming pristine Bi_2_S_3_. Vu *et al.*^78^ fabricated a Bi_2_S_3_@MoS_2_ hierarchical architecture *via* a hydrothermal route. The conformal coating of MoS_2_ nanosheets boosts charge-separation efficiency, increases the number of catalytically active sites, and broadens the light-harvesting window. When the MoS_2_ content reaches 50%, RhB is degraded to 97.5%. The MoS_2_ shell also suppresses Bi_2_S_3_ photocorrosion, so the heterojunction retains its activity after three consecutive cycles with no noticeable decay. The composite thus exhibits both exceptional RhB adsorption and outstanding photocatalytic degradation performance. This design offers a valuable blueprint for developing low-cost and high-performance non-noble-metal cocatalyst systems. Leveraging their core advantages of natural abundance and low price, non-precious-metal cocatalysts have become the leading alternative to noble-metal counterparts. The accumulated studies have achieved significant advances in synthetic protocols, working mechanisms, application scenarios and performance optimization, laying a solid foundation for the industrial transformation of green catalysis technologies.

## 5. Applications of Bi_2_S_3_-based photocatalysts

### 5.1. Photocatalytic degradation of organic pollutants

Owing to its narrow band gap (∼1.3 eV) and deeply positioned CB, Bi_2_S_3_ can generate highly reductive electrons under visible-to-NIR irradiation, making it an ideal candidate for cleaving dye conjugated systems and opening antibiotic rings, thereby achieving full-process reinforcement from rapid adsorption to complete mineralization.

**Fig. 25:**
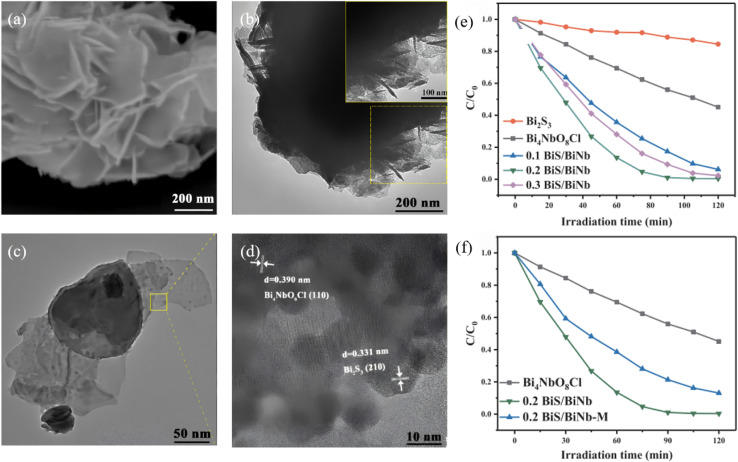
(a and b) SEM, (c) TEM and (d) HRTEM image of 0.2 BiS/BiNb. (e) Photocatalytic degradation efficiencies of RhB with Bi_2_S_3_, Bi_4_NbO_8_Cl, BiS/BiNb hetero-structures under visible-light irradiation. (f) Photocatalytic degradation efficiencies of RhB with Bi_4_NbO_8_Cl, 0.2 BiS/BiNb and 0.2 BiS/BiNb-M under visible-light irradiation.^85^ Copyright 2020, Elsevier.

Qu *et al.*^85^ uniformly anchored Bi_2_S_3_ QDs on Bi_4_NbO_8_Cl nanosheets *via* an *in situ* ion-exchange strategy (Fig. 25). The pronounced quantum-confinement effect and abundant surface active sites extended the absorption edge and accelerated carrier separation. DFT calculations confirmed interfacial electron delocalisation that facilitates charge migration. Under visible light, 100% of RhB was degraded within 90 min, which is far superior to bare Bi_4_NbO_8_Cl or Bi_2_S_3_, and the composite retained its activity after four cycles, demonstrating excellent stability. Holes (h^+^) were identified as the primary active species, assisted by ˙O_2_^−^ and ˙OH radicals. When Bi_2_S_3_ is uniformly dispersed as quantum dots and coupled with Bi_4_NbO_8_Cl to form a heterojunction, a triple synergy emerges: (i) quantum-confinement intensified light absorption, (ii) heterojunction suppressed carrier recombination, and (iii) abundant active sites increase collision probability. Together these effects dramatically accelerate the photodegradation of organic pollutants such as RhB, endowing the composite with distinctive advantages in environmental remediation. Hao *et al.*^86^ prepared Bi_2_S_3_ short nanorods (∼100 nm) by a surfactant-free hydrothermal route and further assembled them with BiOBr nanosheets into a 1-D/2-D Bi_2_S_3_/BiOBr stack. The tight interface and morphology synergy boosted the degradation efficiency to 99.2% for RhB (15 min) and 86.2% for tetracycline (50 min). The enlarged surface area supplied sufficient active adsorption sites, while the green and scalable synthesis offers fresh insight into constructing high-performance sulfide-based heterojunctions. Nisa *et al.*^87^ constructed a SnO_2_/CdSe/Bi_2_S_3_ ternary heterojunction with a stepped band alignment that establishes an efficient electron-transfer highway. Under visible light, 100% MB removal was achieved in 60 min, while 75–99.8% degradation was obtained for RhB, MO and other dyes within the same time-frame. After five cycles, activity remained unchanged along with COD removal reached 91%, confirming effective mineralization. The superior activity originates from the narrow band gap (2.88 eV), multi-interface directional migration and spatial charge separation, which suppress charge recombination and lower charge-transfer resistance.

Collectively, these studies indicate that future Bi_2_S_3_ photocatalysts should prioritise QDs or ultrathin nanosheets as building blocks, while Type-II or stepped-band alignments can be exploited to redirect reaction intermediates, offering a tunable lever for target-specific degradation. Performance optimization has evolved from single-component modification to binary and then to multi-component heterojunction design, with the core scientific logic always centred on efficient separation, migration and utilization of photogenerated charge carriers. Future efforts should focus on atomic-scale engineering of heterointerfaces, *in situ operando* characterization of charge behaviour and pollutant degradation pathways, and the long-term stability and universality of catalysts under real and complex aquatic environments.

### 5.2. Photocatalytic reduction of heavy-metal ions

Among toxic aqueous contaminants, hexavalent chromium (Cr(vi)) is priority owing to its high mobility and proven carcinogenicity. Photocatalytic reduction of Cr(vi) to trivalent chromium (Cr(iii)), which represents a species that readily precipitates as Cr(OH)_3_, offers an energy-efficient remediation route. Bi_2_S_3_ possesses a narrow band gap (∼1.3 eV) and strong visible-light harvesting, yet suffers from rapid electron–hole recombination.

**Fig. 26:**
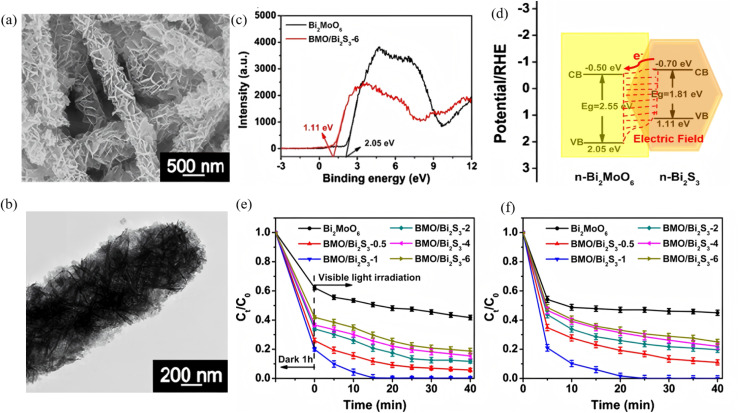
(a) SEM and (b) TEM images of BMO/Bi_2_S_3_-1 heterojunction. (c) XPS VB spectra of pure Bi_2_MoO_6_ and BMO/Bi_2_S_3_-6. (d) Corresponding band structure of the BMO/Bi_2_S_3_-1 heterojunction. (e) Normal photocatalytic Cr(vi) reduction over different samples under visible light. (f) Direct photocatalytic reduction of Cr(vi) without dark treatment.^88^ Copyright 2020, Elsevier.

Constructing heterojunctions, elemental doping and morphology engineering have therefore been widely adopted to accelerate interfacial charge transfer. Li *et al.*^88^ fabricated a Bi_2_MoO_6_ (BMO)/Bi_2_S_3_ framework *via* anion-exchange on MoO_3_ nanobelts (Fig. 26). The open architecture increases specific surface area and shortens charge-diffusion paths. Optimal BMO/Bi_2_S_3_ removes 100% of Cr(vi) within 15 min under visible light, with a rate constant 27.3 times higher than that of bare BMO. Photogenerated electrons are the main active species, assisted by ˙O_2_^−^ radicals. The composite retains its activity after five cycles, confirming high stability. Wang H. *et al.*^89^ prepared Bi_2_S_3_/BiVO_4_/TiO_2_ (BVT) films by *in situ*/*ex situ* methods. In the *in situ* prepared sample, Bi_2_S_3_ nanoribbons intimately contact BiVO_4_/TiO_2_, extending absorption into the visible region and increasing photocurrent density. Citric acid is oxidized by holes in the BiVO_4_ VB, while electrons in the Bi_2_S_3_ CB reduce Cr(vi). The *in situ* BVT achieves 93.9% Cr(vi) reduction in 100 min, which is 14.6 times faster than bare TiO_2_, and maintains ∼70% activity after four cycles. Wang *et al.*^90^ vacuum-synthesized Bi_2_S_3_/Bi heterojunctions in which metallic Bi nanoparticles form Schottky contacts with Bi_2_S_3_. The metal phase acts as an electron mediator and additional Cr(vi) adsorption site. Carrier lifetime increases from 3.8 ns to 10.2 ns, and the reduction rate constant exceeds that of pristine Bi_2_S_3_ by *a* factor of 5.2. Wang *et al.*^91^ reported the preparation of C-doped BiOCl/Bi_2_S_3_ composite, in which the carbon insertion raises the conductivity and optimizes the band alignment. Photoelectrochemical detection of Cr(vi) over this catalyst exhibits a linear range of 0.02–80 µM with a detection limit of 16 nM; at pH 4, 99.5% of Cr(vi) is removed within 120 min, 336 times faster than bare BiOCl. Chahkandi *et al.*^92^ deposited Bi_2_S_3_ films on stainless-steel mesh by square-wave voltammetry, generating nano to micro-scale pores that enhance light harvesting and multiple scattering. Under solar light irradiation, single-component Cr(vi) is completely reduced within 100 min in a Cr(vi)/RhB binary system, in which hole capture by RhB suppresses charge recombination, enabling >93% simultaneous removal of both pollutants with good cyclic stability.

In summary, Bi_2_S_3_-based composite systems can markedly overcome the intrinsic shortcomings of pristine Bi_2_S_3_ for Cr(vi) reduction by constructing heterojunctions, doping with foreign elements, and optimizing synthetic protocols. These strategies effectively promote the separation and migration of photogenerated charge carriers, thereby greatly enhancing both the activity and stability of Bi_2_S_3_-based composites under visible-light irradiation. The key lies in a CB position that lies below the Cr(vi)/Cr(iii) redox potential and in rapid interfacial electron transfer, endowing these materials with broad prospects for heavy-metal wastewater treatment.

### 5.3. Photocatalytic H_2_ evolution

Bi_2_S_3_ has emerged as a key material for constructing high-efficiency hydrogen-evolution photocatalysts thanks to its narrow band gap and excellent visible-light harvesting capability. Nevertheless, its intrinsically rapid recombination of photogenerated charge carriers and insufficient chemical stability severely limit its standalone performance. To overcome these bottlenecks, current research focuses mainly on two directions including building heterojunctions and optimizing interfacial charge transfer. Among them, coupling Bi_2_S_3_ with a well-matched semiconductor to form a heterojunction with ideal band alignment is regarded as one of the most effective strategies for promoting efficient charge separation, suppressing recombination, and enhancing stability.

**Fig. 27:**
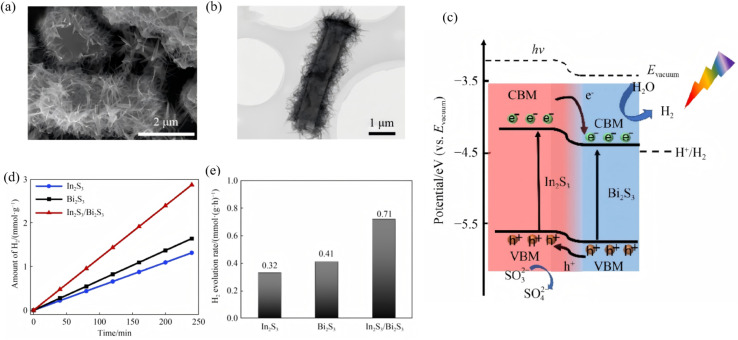
(a) SEM image of In_2_S_3_/Bi_2_S_3_. (b) TEM image of In_2_S_3_/Bi_2_S_3_ heterojunction. (c) Energy level diagram and photocatalytic hydrogen production mechanism of the In_2_S_3_/Bi_2_S_3_. (d) Photocatalytic hydrogen production activity. (e) Photocatalytic hydrogen production rate.^93^ Copyright 2023, Higher Education Press.

Ganapathy *et al.*^77^ constructed a SrTiO_3_/Bi_2_S_3_ heterojunction that exploits the broad-spectral absorption of Bi_2_S_3_ and the well-aligned band positions of SrTiO_3_, markedly promoting the separation and migration of photogenerated carriers and supplying abundant reductive electrons for the photocatalytic hydrogen evolution reaction. Because the SrTiO_3_ CB is more negative (lower in energy), electrons transfer from the SrTiO_3_ CB to the Bi_2_S_3_ CB, forming a Type-I heterojunction. Under UV light, the hydrogen evolution rate of composite is significantly higher than that of pure Bi_2_S_3_, and the composite retains good stability after three cycles. Recently, Liu *et al.*^93^ developed an In-MOF-derived In_2_S_3_/Bi_2_S_3_ heterojunction that further boosts photocatalytic H_2_ evolution (Fig. 27). Rod-like In_2_S_3_ obtained *via* high-temperature sulfidation was decorated *in situ* with flower-like Bi_2_S_3_ grown by a solvothermal method, creating an intimately contacted Type-II heterojunction. The unique architecture not only offers a large specific surface area and abundant active sites, but also enables efficient separation of photogenerated electron–hole pairs through the built-in electric field established at the heterointerface. The built-in electric field drives electrons from the In_2_S_3_ CB to the Bi_2_S_3_ CB for H_2_ evolution, while holes migrate from the Bi_2_S_3_ VB to the In_2_S_3_ VB to react with the sacrificial agent, suppressing carrier recombination and S^2−^ oxidation. The exceptional H_2_-evolution performance of this system is directly ascribed to the synergistic contributions of Bi_2_S_3_'s broad-spectral response, the rapid electron-transfer capability of the MOF-derived In_2_S_3_, and the intimate heterointerface formed between Bi_2_S_3_ and In_2_S_3_, which collectively enhance the photocatalytic activity of the In_2_S_3_/Bi_2_S_3_ composite. This effectively suppresses charge recombination and accelerates interfacial reaction kinetics, offering a fresh strategy for designing highly efficient and stable Bi_2_S_3_-based photocatalytic hydrogen-evolution systems. In terms of morphology and heterojunction construction, it also provides an innovative approach for tailoring unique photocatalysts dedicated to hydrogen production.

### 5.4. Photocatalytic CO_2_ reduction

Since 2019, bismuth-based semiconductors have moved to the forefront of solar-fuel research because of their earth abundance, suitable band positions and visible-light response. Among them, Bi_2_S_3_ possesses a narrow band gap (∼1.3 eV), high absorption coefficient (≈10^5^ cm^−1^) and a CB close to the CO_2_/CH_4_ potential, making it a promising candidate for visible-light-driven CO_2_ reduction.^25^ Stand-alone Bi_2_S_3_, however, suffers from ultrafast electro–hole recombination and a scarcity of surface reductive sites, leading to low selectivity and limited multi-electron kinetics.^94^

**Fig. 28:**
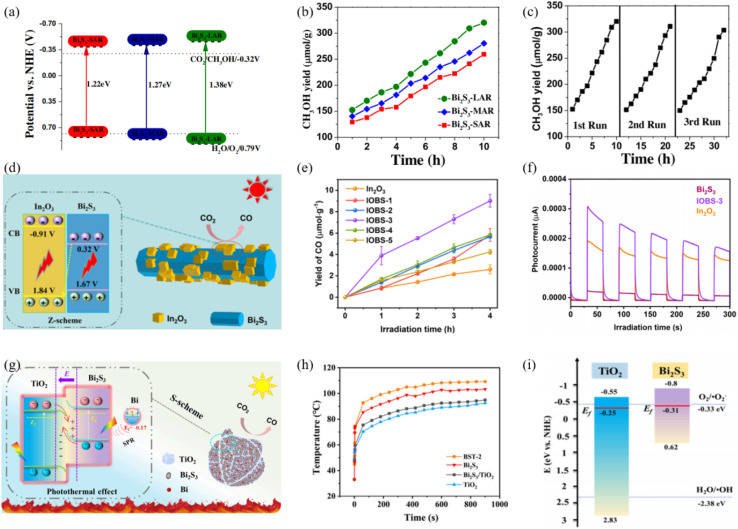
(a) Energy level alignment and bandgap energy of Bi_2_S_3_ together with redox potential of CO_2_/CH_3_OH and H_2_O/O_2_ at pH = 7. (b) Photocatalytic CH_3_OH evolution from Bi_2_S_3_ nanoribbons under 10 h irradiation. (c) Cycling curve of photocatalytic CH_3_OH production over the Bi_2_S_3_ nanoribbons.^96^ Copyright 2017, Elsevier. (d) Schematic illustration of photocatalytic CO_2_ reduction over IOBS-3. (e) CO production curves of In_2_O_3_ and IOBS-*X* along with (f) transient photocurrent responses.^97^ Copyright 2024, American Chemical Society. (g) Diagram of a possible photothermal catalytic mechanism of BST-2 composites. (h) Temperature profiles of various samples under light irradiation. (i) Energy band structure diagram of the prepared samples.^98^ Copyright 2025, American Chemical Society.

Topological transformation of single-crystal Bi_2_O_2_S nanosheets yielded Bi_2_S_3_ nanorods that selectively reduce CO_2_ to CH_4_ under visible light,^95^ while directly synthesized Bi_2_S_3_ nanobelts produce CH_3_OH with enhanced activity^96^ (Fig. 28 a-c). Beyond shape control, heterojunction engineering has become the central strategy. An In_2_O_3_/Bi_2_S_3_ Z-scheme achieves spatial charge separation and delivers CO as the major product^97^ (Fig. 28d–f), whereas a Bi/Bi_2_S_3_/TiO_2_ S-scheme introduces a photothermal-photonic synergy. It significantly enhances CO_2_ reduction performance, proving that constructing heterojunctions is an effective strategy to promote CO_2_ reduction. It is worth considering that future heterojunction designs should go beyond simply choosing Z-type or S-type configurations, and instead focus more on precise control of interface states and a comprehensive analysis of carrier dynamics, in order to prevent the interface from becoming a new center for charge recombination. For example, Bi/Bi_2_S_3_/TiO_2_ system designed by Lu *et al.*, introduced a photothermal synergistic effect^98^ (Fig. 28g–i). It utilizes the LSPR effect of Bi nanoparticles to generate hot electrons, confirming that the photothermal-photonic synergistic mechanism is highly compatible. This study breaks through the limitations of traditional photocatalysis, providing a new pathway to solve critical problems and potentially surpassing the traditional photocatalytic activity limits. It signifies that research is shifting from a pure photon-driven paradigm to a multi-energy-field coupling paradigm, which may be an effective approach to addressing key issues such as low visible light utilization efficiency and slow reaction kinetics.

Despite remarkable progress, Bi_2_S_3_-based CO_2_ photocatalysts still face severe challenges: (i) product selectivity control, (ii) suppression of both bulk and interfacial charge recombination, and (iii) long-term chemical stability under reducing atmospheres.^94^ Future research may necessitate a shift from passive characteriztion to active design. On the one hand, theoretical calculations and *in situ* characterization techniques can be employed to establish structure-activity relationship maps spanning from atomic-level active site structures to macroscopic catalytic performance. On the other hand, rational selection and design of co-catalysts can achieve high selectivity in CO_2_ photocatalytic reduction.^25^ Subsequent research may focus on novel co-catalysts exhibiting enhanced synergistic effects with bismuth substrates, such as single-atom or defect-engineered co-catalysts, to achieve precise adsorption and activation of key reaction intermediates. Only through such synergistic efforts will Bi_2_S_3_-based systems realize efficient, selective and durable solar CO_2_ reduction.

### 5.5. Photoelectrochemical hydrolysis

**Fig. 29:**
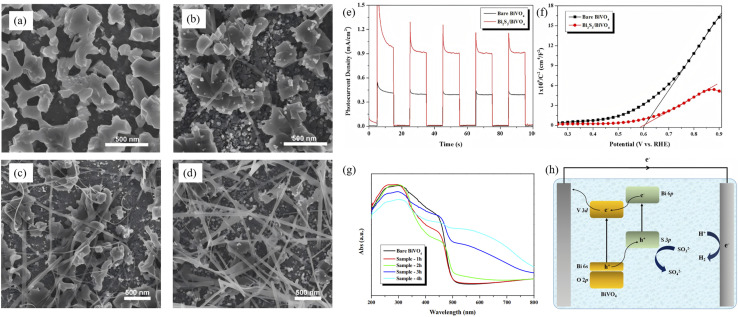
(a–d) SEM images of Bi_2_S_3_/BiVO_4_ hybrid electrodes of (a) sample-1 h; (b) sample-2 h; (c) sample-3 h; and (d) sample-4 h. (e) Photocurrent density and (f) Mott–Schottky plots of the as-prepared photoelectrodes measured in a 0.2 M Na_2_SO_3_ solution (pH = 8). (g) DRS spectra of the bare BiVO_4_ photoelectrode and the Bi_2_S_3_/BiVO_4_ hybrid photoelectrodes with various hydrothermal time. (h) Energy band structure of the hybrid Bi_2_S_3_/BiVO_4_ photoelectrode and charge transfer pathway in the PEC hydrogen production under solar light irradiation.^102^ Copyright 2020, Elsevier BV.

Bi_2_S_3_ films can serve as photoanodes capable of directly converting solar energy into hydrogen energy. Elemental doping and morphology control form the cornerstone for optimizing the intrinsic properties of Bi_2_S_3_ photoanodes, though challenges such as photocorrosion and interfacial charge transport resistance should be effectively addressed. Chalapathi *et al.* systematically investigated the effects of Sb doping^99^ and Cu doping^100^ on the photoelectrochemical (PEC) performance of Bi_2_S_3_ nanorod films. They found that both dopants effectively optimize the electrical properties and band structure of Bi_2_S_3_ nanorod films. Moreover, the Sb^3+^ substitution for Bi^3+^ introduces additional holes, increasing carrier concentration. This significantly enhanced photocurrent density and PEC stability. By modulating the Fermi level and conductivity of material through foreign atoms, this work optimized carrier injection efficiency and setting a new record for Bi_2_S_3_ photoanodes. Furthermore, Bi_2_S_3_ nanorod films grown using seed-layer-assisted techniques exhibit superior crystallinity and tighter substrate contact,^101^ drastically reducing interfacial contact resistance. This provides an efficient pathway for rapid charge transport, maintaining activity without degradation during continuous operation for 4 h, thereby achieving highly efficient photocatalytic water splitting performance. These studies confirm the critical role of interface engineering in enhancing PEC stability. When single-material modifications struggle to simultaneously satisfy all requirements for light absorption, charge separation, and surface reactions, constructing heterojunctions has become an inevitable technical pathway, effectively promoting the separation of photo-generated electron–hole pairs. For instance, coupling Bi_2_S_3_ with BiVO_4_ to form a Bi_2_S_3_/BiVO_4_ heterojunction photoanode^102^ perfectly exemplifies the “complementary advantages” design philosophy (Fig. 29). Bi_2_S_3_ serves as a spectral sensitizer, extending the photoresponse of BiVO_4_ up to 900 nm. The Type-II band alignment drives holes toward the BiVO_4_ VB while electrons travel along the Bi_2_S_3_ CB to the external circuit, enabling bidirectional and ultrafast separation of photogenerated holes and electrons. This synergistic effect is the key to achieving a multiple performance enhancement. Through elemental doping, morphology engineering, and heterojunction construction, the light-harvesting, charge-separation, and charge-transport efficiencies of Bi_2_S_3_-based photoanodes can be markedly enhanced. The critical levers are boosting electrical conductivity, lowering interfacial resistance, and establishing rapid charge-extraction pathways, all of which collectively upgrade their PEC water-splitting performance. Nevertheless, guaranteeing long-term chemical stability remains a central challenge that future research must continuously address and resolve.

### 5.6. Photocatalytic sterilization

The core bactericidal mechanism of Bi_2_S_3_ photocatalysis relies on the irreversible destruction of microbial cell structures by reactive oxygen species (ROS, ˙OH, ˙O_2_^−^, H_2_O_2_) generated through photo-induced charge carriers.^103^ However, the rapid recombination of photogenerated electrons and holes in bare Bi_2_S_3_ limits its disinfection efficiency. Recent research frontiers therefore focus on constructing sophisticated heterojunction systems that synergize multiple sterilization pathways to dramatically boost performance.

**Fig. 30:**
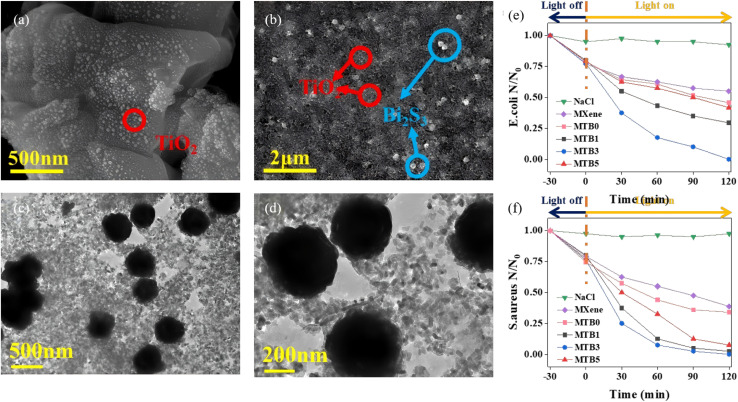
SEM images of (a) MTB0 and (b) MTB3. (c and d) TEM images of MTB3. Photocatalytic sterilization performances of different samples against (e) *E. coli* and (f) *S. aureus* under visible light irradiation.^104^ Copyright 2022, Elsevier.

**Fig. 31:**
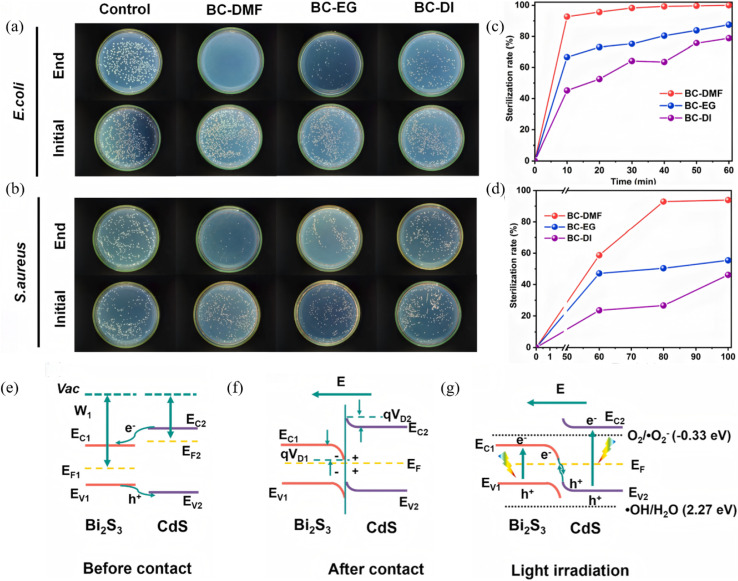
Photographs of (a) *E. coli* and (b) *S. aureus* bacterial colonies sterilized by different photocatalysts under light irradiation under 100 min light irradiation and the effect of irradiation time on antimicrobial behavior against (c) *E. coli* and (d) *S. aureus*. The edge bending at the interface of Bi_2_S_3_/CdS (e) before and (f) after contact. (g) Charge transfer mechanism between Bi_2_S_3_ and CdS under light irradiation.^105^ Copyright 2021, Elsevier.

Constructing a Z-scheme heterojunction is the pivotal strategy for simultaneously optimizing charge separation and preserving strong redox capacity. For instance, a MXene/TiO_2_/Bi_2_S_3_ Z-scheme system was fabricated by a facile hydrothermal oxidation followed by ultrasonic dispersion, anchoring TiO_2_ nanoparticles and Bi_2_S_3_ microspheres uniformly on MXene nanosheets^104^ (Fig. 30). DFT calculations and electron-spin-resonance (ESR) spectroscopy unambiguously confirm that the Z-scheme architecture retains the highly oxidative holes in the TiO_2_ VB (+2.46 eV *vs.* NHE), enabling the generation of ˙OH radicals from H_2_O without any detectable ˙O_2_^−^ radicals. These ˙OH radicals disrupt bacterial cell membranes, diminish enzymatic activity and ultimately arrest cellular metabolism. The apparent reaction-rate constants toward *Escherichia coli* (*E. coli*) and *Staphylococcus aureus* (*S. aureus*) reach 8.4 times and 6.7 times those of pristine MXene, respectively. A Bi_2_S_3_/CdS heterojunction,^105^ prepared in *N*,*N*-dimethylformamide (BC–DMF) was verified by EPR to be a Z-scheme type photocatalyst that generates abundant ˙O_2_^−^ (no ˙OH signal detected) under visible light (Fig. 31). These ˙O_2_^−^ radicals directly attack the *E. coli* membrane, causing intracellular content leakage. That is, a 100% kill rate is achieved within 60 min for *E. coli* and 93.85% within 100 min for *S. aureus*. Beyond efficient spatial separation of photo-carriers, the Z-scheme preserves the strong redox potentials of both holes and electrons, sustaining a high flux of ROS for potent bacterial inactivation and demonstrating the universal applicability of Z-scheme architectures against drug-resistant strains. A prominent current trend is the shift from a single-mechanism paradigm to multi-mechanism synergy. ROS attack alone is often insufficient against biofilms or deep-seated infections. Addressing this, Feng *et al.*,^106^ engineered a BiOI@Bi_2_S_3_/MXene heterostructure *via in situ* sulfidation to create intimate interfacial contact, ingeniously coupling the outstanding photothermal conversion of Bi_2_S_3_ (57.8% efficiency, temperature rising to 86.1 °C under 808 nm irradiation) with the robust photocatalytic activity of the heterojunction. The locally generated heat not only directly disrupts bacterial structures but also accelerates metabolic activity, rendering the bacteria more susceptible to ROS and thereby enabling efficient eradication of drug-resistant strains and biofilms. Meanwhile, the Z-scheme preserves highly oxidative holes that continuously produce ˙OH and H_2_O_2_. Under the synergistic action of photothermal therapy (PTT) and photodynamic therapy (PDT), bactericidal rates against *Pseudomonas aeruginosa* and *S. aureus* reach 99.7% and 99.8%, respectively, while cytotoxicity assays reveal no appreciable damage to mammalian cells.

Therefore, constructing Z-scheme heterojunctions to optimize charge separation while synergizing PTT and PDT effects is an effective strategy for boosting the photocatalytic antimicrobial performance of Bi_2_S_3_-based materials. Future breakthroughs must tackle the central challenges of safety, long-term stability, and adaptability to complex real-world environments that arise during practical deployment.

## 6. Challenges and future prospects

### 6.1. Key challenges at present

#### 6.1.1. Fundamental improvement in photostability

Although Bi_2_S_3_ has demonstrated great potential in various photochemical energy applications, its intrinsic material instability severely constrains practical deployment and commercialization. Foremost among the current challenges is the fundamental improvement of its photostability and chemical stability. The instability of Bi_2_S_3_ is manifested by the photo-oxidation of S^2−^ ions by photogenerated holes under illumination, leading to self-photocorrosion, loss of active components, and rapid performance degradation.^107^ By inserting a hole-intercepting Type-II or Z-scheme heterojunction, the holes are rapidly shuttled to a wide-band-gap oxygen-containing semiconductor (TiO_2_, BiVO_4_), leaving the Bi_2_S_3_ side electron-rich. This was the first quantitative demonstration that a heterojunction can completely block the self-oxidation pathway.^107^ Encapsulating Bi_2_S_3_ with a thin polymer overlayer creates an interfacial passivation layer that serves as a physical barrier: It not only blocks ambient O_2_ and H_2_O from reaching the sulfide surface, thereby suppressing indirect photocorrosion (O_2_ + e^−^ → ˙O_2_^−^, followed by S^2−^ oxidation), but also impedes hole migration to the surface, markedly lowering the probability of S^2−^/hole contact. This molecular-level protective shield thus isolates the material from both oxygen and holes.^108–110^ Alternatively, self-healing can be engineered by creating reversible sulfur vacancies or an external sulfur reservoir that compensates for sulfur loss *in situ*, and recent work has already demonstrated the feasibility of this self-repair concept.

These combined strategies have already cut the photocorrosion rate of Bi_2_S_3_ by an order of magnitude, furnishing a generic stability-design framework that enables narrow-band-gap photocatalysts to operate outdoors for extended periods. Future research must therefore mount a concerted attack, ranging from intrinsic structural stabilization of the material itself to advanced interfacial passivation engineering, to deliver truly definitive solutions.

#### 6.1.2. Further boosting charge-separation efficiency

Despite its exceptional visible-light harvesting capability, Bi_2_S_3_ suffers from an intrinsically high carrier-recombination rate that severely limits the usable fraction of photogenerated electron–hole pairs, constituting a key bottleneck to higher photocatalytic performance. Present research shows that engineering multi-component heterojunctions has become the mainstream route for pushing charge-separation efficiency to the next level.

In recent years, heterojunction design has evolved from simple binary systems toward more sophisticated and multi-functional architectures. Current efforts focus on precise band-alignment and interface engineering to create multi-component heterojunctions such as dual S-scheme or Z-scheme systems with directional charge-transfer pathways that maximize the separation and utilization of photogenerated carriers. Harnessing tunable multi-component heterojunctions, integrating synergistic mechanisms, and deploying *operando* characterization technology collectively constitute an effective approach to overcoming the inherent charge separation limitations of Bi_2_S_3_. Yet this progress introduces fresh challenges, that is, as junction architectures grow increasingly complex, *in situ* tracking and precise control of interfacial charge-transfer pathways become ever more demanding. Future efforts must therefore delve deeper into the charge-kinetics of these multinary systems, striving to maximize separation efficiency while simultaneously minimizing interfacial transport resistance, which is an essential step toward a fundamental breakthrough in charge-utilization efficiency.

### 6.2. Future research directions

To break the fundamental limits of Bi_2_S_3_-based materials in both efficiency and stability, the next frontier is to assemble ternary or even higher-order heterojunction architectures in which multiple mechanisms cooperate in light harvesting, charge separation, and surface reaction. Rather than solving one bottleneck at a time, future designs must choreograph carrier generation, separation, transport, and utilization within a single platform. This demands the seamless integration of elemental doping, heterojunction engineering, surface functionalization, and external-field effects (*e.g.*, photothermal, piezoelectric, plasmonic, *etc.*). By coupling and amplifying these multi-mechanistic and multi-functional roles, Bi_2_S_3_-based artificial photosystems can be propelled toward step-change advances in energy, environmental, and optoelectronic applications.

## 7. Conclusions

This review summarizes the research progress of Bi_2_S_3_-based photocatalysts from 2019 to 2025, systematically collating their fundamental properties and controllable syntheses, and further clarifying optimization strategies. It then surveys their practical applications, charting a path from traditional pollutant degradation to CO_2_ reduction, N_2_ fixation, PEC H_2_ evolution and antibacterial use, realizing high-value, multi-scenario deployment. Bi_2_S_3_-based photocatalysis has already leapt from material exploration to large performance gains, and thus future work must uncover performance truths at the atomic and electronic scale, dissect synergistic mechanisms with atomic precision, and finally transform Bi_2_S_3_ photocatalysts from “paper results” into “real-world products.” toward high-efficiency solar energy conversion.

## Author contributions

Wei Zhao and Qing Chen wrote the manuscript and they contributed equally to this work. Jie Liang provided helps for organizing the graphs. Lifeng Cai and Fang-Xing Xiao guided this work and helped correct the manuscript.

## Conflicts of interest

There are no conflicts to declare.

## Data Availability

No primary research results, software or code have been included and no new data were generated or analysed as part of this review.

## References

[cit1] Xu F. H., Weng B. C. (2023). J. Mater. Chem. A.

[cit2] Yaghoubi S., Mousavi S. M., Babapoor A., Binazadeh M., Lai C. W., Althomali R. H., Rahman M. M., Chiang W. H. (2024). Renew. Sustain. Energy Rev..

[cit3] Huang Y., Zhang J. F., Ruzimuradov O., Mamatkulov S., Dai K., Low J. X. (2025). Compos. Funct. Mater..

[cit4] Shi Y., Yang A. F., Cao C. S., Zhao B. (2019). Coord. Chem. Rev..

[cit5] Li T. Y., Wang P., He M., Zhang T. B., Yang C., Li Z. X. (2024). Coord. Chem. Rev..

[cit6] Li K., Gao Y., Dong Z. X., Zhang H. B., Fan X. D., Xu L., Huang J., Teng F., Fan H. B., Song J. M., Zhang C. M., He X. X., Hu P. (2024). Environ. Res..

[cit7] Zhao H. Y., Wang S., Zhu H. Y., Zhang X. X., Shang D. H., Zhou X. W., Wang J., Zhu C. Z., Du F., Song Y. Y., Yang F. (2024). Rare Met..

[cit8] Maeda K., Domen K. (2010). J. Phys. Chem. Lett..

[cit9] Arora I., Chawla H., Chandra A., Sagadevan S., Garg S. (2022). Inorg. Chem. Commun..

[cit10] Sienkiewicz A., Wanag A., Kusiak-Nejman E., Ekiert E., Rokicka-Konieczna P., Morawski A. W. (2021). J. Environ. Chem. Eng..

[cit11] Chen Y., Li A., Fu X. L., Peng Z. J. (2023). Appl. Surf. Sci..

[cit12] Huang D. H., Wu T. F., Xie D. Y., Che H. N., Ao Y. H. (2025). Compos. Funct. Mater..

[cit13] Park H., Park Y., Kim W., Choi W. (2013). J. Photochem. Photobiol. C: Photochem. Rev..

[cit14] Tang J. Y., Pang J. Y., Lv X. X., Wang X. L. (2025). ACS Appl. Energy Mater..

[cit15] Zhang L., Ai Z. Z., Xu X. L., Shi D., Zhang B. G., Hu H. X., Yang M. Z., Shao Y. L., Wu Y. Z., Hao X. P. (2023). Ionics.

[cit16] Chawla A., Sudhaik A., Kumar R., Raizada P., Ahamad T., Khan A. A. P., Le Q. V., Nguyen V., Thakur S., Singh P. (2025). Coord. Chem. Rev..

[cit17] Cao W., Wang M., Yang J., Han B., Zhu X. C., Wang Y. P. (2022). J. Solid State Chem..

[cit18] Li J. P., Wang B., Wang T., Zhao Y., Song T., Zhang L. N., Cheng X. (2020). J. Alloys Compd..

[cit19] Wang J. H., Yang Y., Ye X. J., Ren W., Li L., Zheng X. Z., Ge J. B., Zhang S. J., Chen S. F. (2025). J. Am. Ceram. Soc..

[cit20] Xing B., Wang T., Han X. B., Zhang K., Li B. X. (2023). J. Colloid Interface Sci..

[cit21] Yan K., Wu D. H., Wang T., Chen C., Liu S. J., Hu Y. G., Gao C., Chen H. Y., Li B. X. (2023). ACS Catal..

[cit22] Miodynska M., Mikolajczyk A., Bajorowicz B., Zwara J., Klimczuk T., Lisowski W., Trykowski G., Pinto H. P., Zaleska-Medynska A. (2020). Appl. Catal. B: Environ..

[cit23] Jia Q. F., Li M., Sun W. J. (2025). Dalton Trans..

[cit24] Shen P., Li N., Nasser A. M., Zhu B., Xi X., She L. J., Liu Y. F., Ma J. Q. (2024). Langmuir.

[cit25] Li X., Yu J. G., Jaroniec M., Chen X. B. (2019). Chem. Rev..

[cit26] Xing X., Zhang L. X., Ren Y., Li Y. F., Yu H., Shi W. W. (2024). J. Environ. Chem. Eng..

[cit27] Kyono A., Kimata M. (2004). Am. Mineral..

[cit28] Hao Q., Xie C., Huang Y. M., Chen D. M., Liu Y. W., Wei W., Ni B. J. (2020). Chin. J. Catal..

[cit29] Al Anazi A. A., Treve M., Ali A., Albaker A., Kareem A. K., Jain S., Altamimi A. S., Romero-Parra R. M., Al-Kharsan I. H., Alhassan M. S. (2023). Mater. Res. Bull..

[cit30] McKeehan L. W. (1923). J. Frankl. Inst..

[cit31] Zhang H., Wang L. J. (2007). Mater. Lett..

[cit32] Deshpande M. P., Sakariya P. N., Bhatt S. V., Garg N., Patel K., Chaki S. H. (2014). Mater. Sci. Semicond. Process..

[cit33] Escoda-Torroella M., Moya C., Ruiz-Torres J. A., Rodríguez A. F., Labarta A., Batlle X. (2023). Phys. Chem. Chem. Phys..

[cit34] Zahedi E. (2015). Superlattices Microstruct..

[cit35] Yan W., Chen X., Wang Z. Q., Zhao Z. Y., Liu Y., Muhammad A. (2025). Chem. Eng. J..

[cit36] Yi D., Chen X., Cai W. F., Li L. C. (2025). Surf. Sci..

[cit37] Chen J. S., Qin S. Y., Song G. X., Xiang T. Y., Xin F., Yin X. H. (2013). Dalton Trans..

[cit38] Li H., Wang X. T., Wei Q. Y., Hou B. R. (2017). Nanoscale Res. Lett..

[cit39] Chen Y. T., Xia W. X., Zhou Y., Zhang Q. R., Chen X. B., Ma L., Ding S. J. (2025). J. Environ. Chem. Eng..

[cit40] Jiang J., Che X., Qian Y. W., Wang L. Z. Y., Zhang Y., Wang Z. L. (2020). Front. Mater..

[cit41] Joy R., Meena B., Kumar M., Joseph M., Joseph S., Subrahmanyam C., Haridas S. (2024). Catal. Today.

[cit42] Xiao Y. W., Li M. Y., Li H. Y., Wang Z. Z., Wang Y. D. (2024). Nano Energy.

[cit43] Liu Z. P., Peng S., Xie Q., Hu Z. K., Yang Y., Zhang S. Y., Qian Y. T. (2003). Adv. Mater..

[cit44] Yang L. J., Hu Y. D., Zhang L. (2019). Chem. Eng. J..

[cit45] Sang Y., Cao X., Dai G. D., Wang L. X., Peng Y., Geng B. Y. (2020). J. Hazard. Mater..

[cit46] Wang L. J., Karuturi S., Zan L. (2021). Small.

[cit47] Wu T., Zhou X. G., Zhang H., Zhong X. H. (2010). Nano Res..

[cit48] Saah S. A., Afzaal M., O’Brien P. (2022). Results Chem..

[cit49] Lu Y., Song J. M., Li W. F., Pan Y. L., Fang H. Y., Wang X. L., Hu G. (2020). Appl. Surf. Sci..

[cit50] Huang H. B., Zhang N., Xu J. Y., Xu Y. H., Li Y. F., Lü J., Cao R. (2022). Research.

[cit51] Xu F., Xu C. Y., Chen H. M., Wu D. P., Gao Z. Y., Ma X. M., Zhang Q., Jiang K. (2019). J. Alloys Compd..

[cit52] Li W. H. (2008). Mater. Lett..

[cit53] Godzierz M., Mistewicz K., Gawron A., Kurtyka K., Otulakowski L., Das T. K. (2024). J. Alloys Compd..

[cit54] Dai D. L., Qiu J. H., Xia G. L., Tang Y., Liu Q. Y., Li Y. X., Fang B. Y., Yao J. F. (2024). Small.

[cit55] Mi Y. W., Li H. P., Yu X., Zhang Y. F., Zeng S. Y., Wang L., Hou W. G. (2024). Appl. Surf. Sci..

[cit56] Uddin I., Abzal S. M., Kalyan K., Janga S., Rath A., Patel R., Gupta D. K., Ravindran T. R., Ateeq H., Khan M. S., Dash J. K. (2022). ACS Omega.

[cit57] Li Y. P., Chen J. L., Chen S., Lu T. T., Liao X. L., Zhao T. T., Cheng F. Y., Wang H. (2020). Mater. Rep..

[cit58] Liu C. J., Yang Y., Li W. Z., Li J., Li Y. M., Chen Q. Y. (2016). Chem. Eng. J..

[cit59] Arumugam J., George A., Venci X., Raj A. D., Irudayaraj A. A., Josphine R. L., Sundaram S. J., Al-onazi W. A., Al-Mohaimeed A. M., Chen T. W., Kaviyarasu K. (2022). J. Alloys Compd..

[cit60] Messalea K. A., Zavabeti A., Mohiuddin M., Syed N., Jannat A., Atkin P., Ahmed T., Walia S., McConville C. F., Kalantar-Zadeh K., Mahmood N., Khoshmanesh K., Daeneke T. (2020). Adv. Mater. Interfaces.

[cit61] Li Y. Z., Chen J. L., Chen S., Lu T. T., Liao X. L., Zhao T. T., Cheng F. Y., Wang H. (2024). Appl. Catal. B: Environ. Energy.

[cit62] Zhou R. T., Tu X. M., Zheng P., Zhang L., Zeng Z. X. (2023). Molecules.

[cit63] Lian X. Y., Zhang J. G., Zhan Y., Zhang Y. P., Yang S. L., Chen Z., Dong Y. Y., Fang W. P., Yi X. D. (2021). J. Hazard. Mater..

[cit64] Dang C. C., He S. X., Liu Y. P., Zhao L. C., Shan A. D., Li M., Kong L. T., Gao L. M. (2023). Chem. Eng. J..

[cit65] Yuan Z. H., Tuerhong M., Aisikaer X., Mamtmin G. (2025). Appl. Organomet. Chem..

[cit66] Fan X. Y., Liang H. O., Zhang M., Li C. P., Bai J. (2025). Colloids Surf. A: Physicochem. Eng. Aspects.

[cit67] Guo Z. Z., Ren Z. J., Gao H. M., Guan J. F., Zheng R. J., Li P. Y. (2024). Appl. Surf. Sci..

[cit68] Chachvalvutikul A., Pudkon W., Luangwanta T., Thongtem T., Thongtem S., Kittiwachana S., Kaowphong S. (2019). Mater. Res. Bull..

[cit69] Ke J., Liu J., Sun H. Q., Zhang H. Y., Duan X. G., Liang P., Li X. Y., Tade M. O., Liu S. M., Wang S. B. (2017). Appl. Catal. B: Environ. Energy.

[cit70] Latifian P., Hosseini S. F., Dorraji M. S. S., Rasoulifard M. H. (2023). J. Mol. Liq..

[cit71] Hosseini S. F., Dorraji M. S. S., Rasoulifard M. H. (2023). Compos. Part B: Eng..

[cit72] Sun Y. X., Li J. H., Wang Z. Y., Zhu H. C. (2025). J. Mater. Chem. A.

[cit73] Zhou W., Li Y., Huang H. X., Wang J. Y., Zhong F. X. (2022). Mater. Sci. Semicond. Process..

[cit74] Nkwe V. M., Olatunde O. C., Ben Smida Y., Siddeeg S. M., Onwudiwe D. C. (2023). Mater. Today Commun..

[cit75] Du F. Y., Lai Z., Tang H. Y., Wang H. Y., Zhao C. X. (2022). Chemosphere.

[cit76] Shi X. D., Qin X. Y., Yang X. Y., Wei X. Y., Liu Y., Li S. H., Liu G. X., Wang J. X., Dong X. T., Chen F. H. (2024). Mater. Today Chem..

[cit77] Ganapathy M., Hsu Y., Thomas J., Chen L. Y., Chang C. T., Alagan V. (2021). Energy Fuels.

[cit78] Vu T. T. H., Do T. A. T., Nguyen D. T., Ho T. G., Pham Q. N., Giang H. T., Hoang M. H., Nghiem T. H., Man M. T., Tran D. L. (2022). Results Mater.

[cit79] Zha R. H., Niu Y. H., Liu C. Y., He L., Zhang M. (2021). J. Environ. Chem. Eng..

[cit80] Lan M., Dong X. L., Zheng N., Zhang X. X., Wang Y., Zhang X. X. (2023). J. Mater. Sci. Technol..

[cit81] Lan M., Dong X. L., Zheng N., Zhang Y. B. (2025). Chem. Eng. Sci..

[cit82] Zhu Y. J., Han Q. T., Qu H., Shen Y., Zhou X., Zou Z. G., Zhou Y. (2024). Catal. Sci. Technol..

[cit83] Nwaji N., Akinoglu E. M., Giersig M. (2021). Catalysts.

[cit84] Liu H. Y., Wu R., Tian L., Kong Y. Y., Fan H. M., Yang X., Sun Y. F. (2019). Mater. Lett..

[cit85] Qu X. F., Gao Z. Q., Liu M. H., Zhai H. J., Shi L., Li Y., Song H. B. (2020). Appl. Surf. Sci..

[cit86] Hao T. R., Xu H. L., Yu H., Li M. L., Song B., Shao G., Fan B. B., Wang H. L., Lu H. X., Zhang R. (2025). Sep. Purif. Technol..

[cit87] Nisa M. U., Abid A. G., Gouadria S., Munawar T., Alrowaili Z. A., Abdullah M., Al-Buriahi M. S., Iqbal F., Ehsan M. F., Ashiq M. N. (2022). Surf. Interfaces.

[cit88] Li X. Q., Chen D. Y., Li N. J., Xu Q. F., Li H., He J. H., Lu J. M. (2020). J. Hazard. Mater..

[cit89] Wang H. W., Zhu Y. W., Joshi M. K., Cheng Y., Zhang P. Y., Tan M. H., Yu R. H., Mao Z. P., Li X. L. (2024). Chem. Eng. J..

[cit90] Wang X. Q., Wang F., Xu B. Q., Yang B. (2025). Appl. Surf. Sci..

[cit91] Wang C. L., Liu N. Z., Zhao X., Tian Y., Chen X. W., Zhang Y. F., Fan L., Hou B. R. (2023). J. Mater. Sci. Technol..

[cit92] Chahkandi M., Zargazi M. (2019). J. Hazard. Mater..

[cit93] Liu S. B., Wang Y. J., Zhang Y. Z., Xin X., Guo P., Deng D. S., Ghasemi J. B., Wang M., Wang R. L., Li X. H. (2023). Front. Energy.

[cit94] Liao G., Wang H., Zhang J. (2024). Precis. Chem..

[cit95] Jiang L. S., Hu Y., Wang K., Liang C., Liu C. Y., Li X. Q., Jia Y. Y., Liu W. (2023). Sep. Purif. Technol..

[cit96] Jin J. R., He T. (2017). Appl. Surf. Sci..

[cit97] Sun M. F., Fan K., Liu C. Y., Gui T., Dai C. H., Jia Y. S., Liu X., Zeng C. (2024). Langmuir.

[cit98] Lu M. H., Zhang K., Peng D. M., Luo M. Y., Zhang Y., Qin Y. M. (2025). Langmuir.

[cit99] Chalapathi U., Reddy N. P., Alhammadi S., Alshgari R. A., Dhanalakshmi R., Reddy G. S., Sangaraju S., Mohanarangam K., Reddy V. R. M., Ahn C. H., Park S. H. (2025). J. Solid State Chem..

[cit100] Chalapathi U., Cuddapah D. R., Reddy P. B., Alhammadi S., Alshgari R. A., Radhalayam D., Golkonda S. R., Sangaraju S., Mohanarangam K., Reddy V., Park S. H. (2024). Opt. Mater..

[cit101] Chalapathi U., Reddy B. P., Dhanalakshmi R., Reddy G. S., Divya A., Sangaraju S., Alhammadi S., Mohanarangam K., Bahajjaj A. A. A., Park S. H. (2025). Int. J. Hydrogen Energy.

[cit102] Li F., Leung D. Y. C. (2020). Chem. Eng. Sci..

[cit103] Ge L. F., Wang W., Tan F. T., Wang X. Y., Qiao X. L., Wong P. K. (2023). Sol. RRL.

[cit104] Huang H. M., Zhang J. F., Tang C. M., Li A. Y., Zhang T. M., Xue H. P., Zhang D. F. (2022). J. Environ. Chem. Eng..

[cit105] Shi L. Y., Ma Z. L., Qu W. W., Zhou W., Deng Z. Q., Zhang H. F. (2021). Appl. Surf. Sci..

[cit106] Feng H. M., Wang W., Wang T., Pu Y. A., Ma C. C., Chen S. G. (2023). Acta Biomater..

[cit107] Yan Y. H., Zhou Z. X., Li W. Q., Zhu Y. J., Cheng Y., Zhao F. Y., Zhou J. G. (2014). RSC Adv..

[cit108] Guo Z. P., Wei W., Li Y. H., Li Z. Y., Hou F. M., Wei A. (2022). J. Hazard. Mater..

[cit109] Sun Y., Liu C., Ji S., Ni J., Wu X., Silva S. R. P., Cai M., Shao G., Zhang P. (2025). Compos. Funct. Mater..

[cit110] Zhang H., Fan Y., Guan S., Cui W.-G., Zhang M., Li Z., Dou Y., Yang J., Zhuang Z., Yuan Z., Zhao S., Wang D., Liu B., Pan H. (2025). Compos. Funct. Mater..

